# Insights on the bacterial composition of Parmigiano Reggiano Natural Whey Starter by a culture-dependent and 16S rRNA metabarcoding portrait

**DOI:** 10.1038/s41598-022-22207-y

**Published:** 2022-10-15

**Authors:** Laura Sola, Emanuele Quadu, Elena Bortolazzo, Loris Bertoldi, Cinzia L. Randazzo, Valentina Pizzamiglio, Lisa Solieri

**Affiliations:** 1 Department of Life Sciences, University of Modena and Reggio Emilia, 42122 Reggio Emilia, Italy; 2 Centro Ricerche Produzioni Animali, 42121 Reggio Emilia, Italy; 3 BMR Genomics Srl, 35131 Padua, Italy; 4 Department of Agriculture, Food and Environment, University of Catania, 95123 Catania, Italy; 5 ProBioEtna Srl, 95123 Catania, Italy; 6 Consorzio del Formaggio Parmigiano Reggiano, 42124 Reggio Emilia, Italy; 7 NBFC, National Biodiversity Future Center, 90133 Palermo, Italy

**Keywords:** Environmental microbiology, Microbial ecology, Microbiome

## Abstract

Natural whey starters (NWS) are undefined bacterial communities produced daily from whey of the previous cheese-making round, by application of high temperature. As a result, in any dairy plant, NWS are continuously evolving, undefined mixtures of several strains and/or species of lactic acid bacteria, whose composition and performance strongly depend on the selective pressure acting during incubation. While NWS is critical to assure consistency to cheese-making process, little is known about the composition, functional features, and plant-to-plant fluctuations. Here, we integrated 16S rRNA metabarcoding and culture-dependent methods to profile bacterial communities of 10 NWS sampled in the production area of Parmigiano Reggiano cheese. 16S rRNA metabarcoding analysis revealed two main NWS community types, namely NWS type-H and NWS type-D. *Lactobacillus helveticus* was more abundant in NWS type-H, whilst *Lactobacillus delbrueckii*/*St. thermophilus* in NWS type-D, respectively. Based on the prediction of metagenome functions, NWS type-H samples were enriched in functional pathways related to galactose catabolism and purine metabolism, while NWS type-D in pathways related to aromatic and branched chain amino acid biosynthesis, which are flavor compound precursors. Culture-dependent approaches revealed low cultivability of individual colonies as axenic cultures and high genetic diversity in the pool of cultivable survivors. Co-culturing experiments showed that fermentative performance decreases by reducing the bacterial complexity of inoculum, suggesting that biotic interactions and cross-feeding relationships could take place in NWS communities, assuring phenotypic robustness. Even though our data cannot directly predict these ecological interactions, this study provides the basis for experiments targeted at understanding how selective regime affects composition, bacterial interaction, and fermentative performance in NWS.

## Introduction

Conventionally, two different types of starter cultures are being exploited in industrialized cheese production: defined starter cultures and complex undefined starter cultures^1^. Defined cheese starter cultures are usually composed of one or more strains of mesophilic lactococci with known characteristics. These individual strains have been generally isolated from undefined complex starter cultures to obtain axenic single strains. The currently used undefined starter cultures were generally isolated in the 50s and 60s of the twentieth century from cheese production farms and were kept frozen to retain their original composition. For example, cultures used in the Netherlands to produce Gouda cheese are domesticated undefined cultures stored as frozen stocks and reactivated only the strictly necessary to minimize the numbers of propagation cycles and to limit compositional shifts^2^. Both defined and undefined starter cultures are essential for fermenting lactose into lactate and degrading caseins, resulting in the milk acidification and preservation^3^. However, mixed-strains undefined starter cultures were reported to be more resilient to stress and bacteriophage attack and to display a more robust performance compared with defined low-strain-diversity dairy cultures^4^. These properties have triggered a renaissance of interest towards undefined starter cultures with the aim to understand bacterial interactions and co-evolution forces shaping these communities^5–9^.

In Italian long ripened hard cheese production as for Parmigiano Reggiano, Grana Padano, and Trentingrana, a third kind of undefined starter cultures is produced by incubating cheese whey under conditions that favor the growth of desirable thermophilic lactic acid bacteria (LAB)^10^. In case of Parmigiano Reggiano (PR) cheese, natural whey starter (NWS) is daily produced in every farm belonging to the Protected Designation of Origin (PDO) Consortium (Specifications of Parmigiano Reggiano Cheese; https://www.parmigianoreggiano.com/consortium/rules_regulation_2/default.aspx). A part of the whey of the previous cheesemaking round is removed after curd cooking (54–56 °C), progressively cooled until reaching 49–46 °C for approximately 20 h, and subsequently used to inoculate a new milk batch for the following cheese production. Consequently, NWS cultures encounter considerable environmental changes and consist of a highly variable bacterial communities which differ over time and space in species and strain composition depending upon the trend in whey cooling, the size of fermentation bioreactor, and the bacterial quality of cheese whey. The PR NWS variability is further increased by the usage of unpasteurized cow milk as raw material which in turn affects the microbiota inhabiting cheese whey. Previous studies on PR NWS attributed predominant microbiota to *Lactobacillus helveticus*^11–14^. Further knowledge on bacterial composition of PR NWS was provided by Bottari et al.^15^ and Bertani et al.^16^ by using culture-independent techniques. These seminal studies demonstrated that PR NWS is composed by comparable percentages of *L. helveticus* and *Lactobacillus delbrueckii*, while *Limosilactobacillus fermentum* (basionym: *Lactobacillus fermentum*) and *Streptococcus thermophilus* are present to a lesser extent. All these species are acid-tolerant, microaerophilic, thermophilic, and well adapted to thrive the selection pressure occurring during whey fermentation^17–19^.

Metabarcoding approaches, based on high throughput sequencing (HST) of variable regions of the 16S rRNA gene, have been widely used to describe the composition of bacterial communities in dairy ecosystems, generating a body of knowledge useful to improve quality and safety of dairy products^20,21^. Although the NWS performance is critical to assure reliability of PR cheesemaking, there is limited information on the NWS variability in term of species abundance over the PDO production area. Yet, we know little about the microbial interactions inside PR NWS which can shape microbiota composition.

The aim of this pilot study was to provide a culture dependent and independent profile of microbiota inhabiting PR NWS sampled over the PDO production area and to correlate them with physicochemical and technological parameters.

## Results

### Physicochemical parameters and viable microbial counts

**Figure 1:**
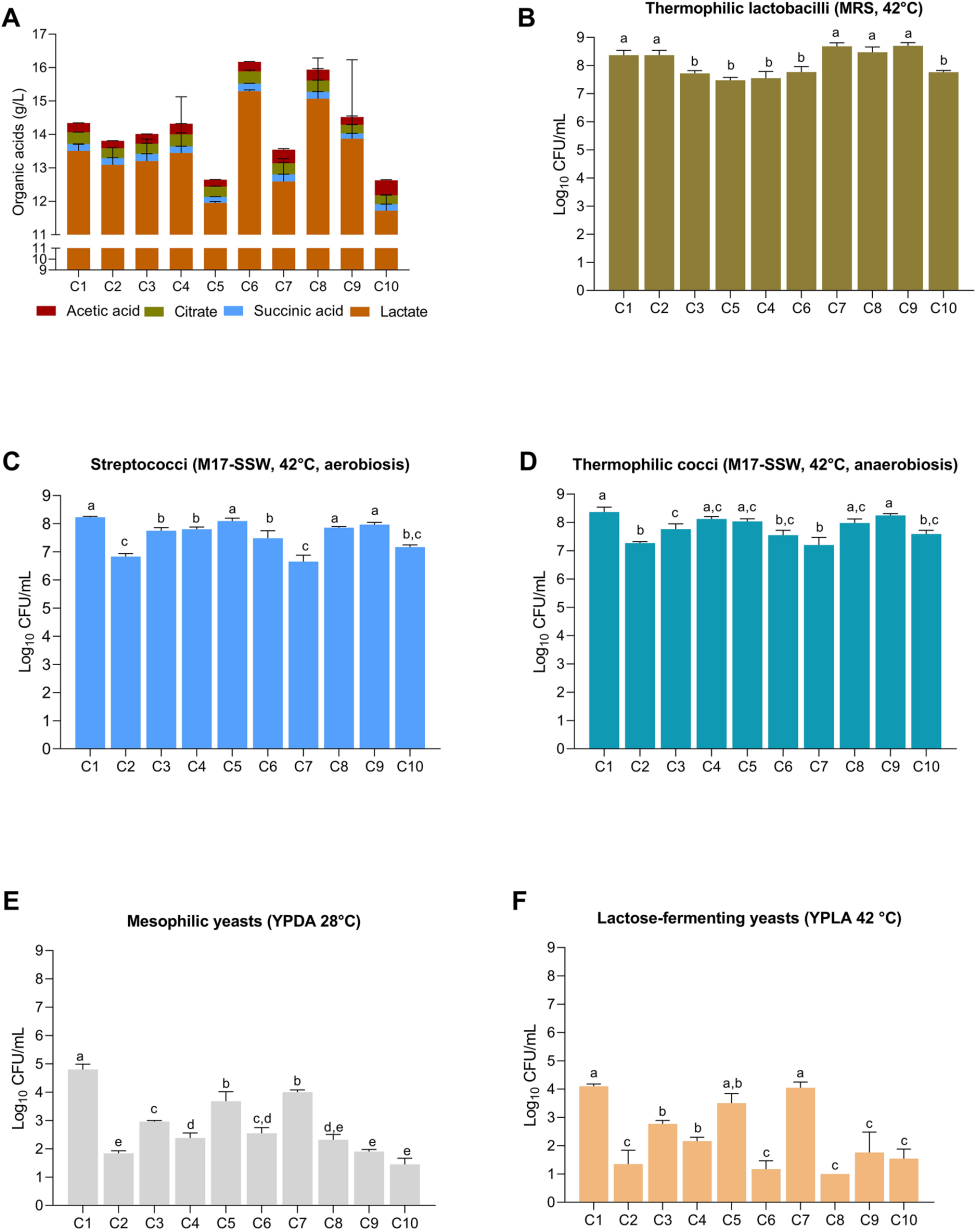
Organic acids contents and microbial counts of ten NWS samples. Organic acids concentrations are expressed in g/L as means ± standard deviation of four replicates (**A**). Microbial counts of presumptive lactobacilli (**B**), streptococci (**C**), cocci-shaped LAB (**D**), mesophilic yeasts (**E**), and lactose-fermenting thermophilic yeasts (**F**) are expressed as means of Log_10_ CFU/mL of at least three replicates. Significant differences are indicated with different letters (*p* < 0.05), as calculated by one-way ANOVA. Plotted with GraphPad Prism v.8.00 software (San Diego, CA, USA, https://www.graphpad.com/).

Ten NWS samples, termed C1 to C10, were collected from different dairy farms located in the production area of PR cheese during the Autumn. Values of pH ranged from 3.27 to 3.57, while titratable acidity was from 27.3 to 33.4 SH°/50 mL (Supplementary Table S1). Fermentative performance of NWS samples, measured as ΔSH°/50 mL, were also highly variable, suggesting differences among NWS samples in milk acidification rate. As expected, lactate was the main organic acid detected in NWS (Fig. 1A), but its content was variable among samples, reflecting differences in NWS microbial composition according to the home-made nature of mixed microbiota forming NWS (Supplementary Table S1). Lactate concentrations in samples C5 and C10 were significantly lower than in other NWS (*p* < 0.05). During lactic fermentation, some organic acids (lactic and acetic acid) increase, while other organic acids (citric acid, etc.) derived from lipolysis, carbohydrate metabolism, or amino acid metabolism decrease^22^. Under low pH conditions, homofermentative species could undergo a shift from homofermentative to a mixed-acid profile with the production of acetate and succinate from citrate catabolism^23^. Furthermore, citric, acetic, and succinic acids were also by-products of yeast sugar catabolism. Citric acid was the second organic acid present in the NWS analyzed, followed by acetic and succinic acids. Sample C10 showed the highest concentration of acetic acid (*p* > 0.05), followed by C7 and C8. The non-dissociated form of acetic acid is dependent on the external pH and on the pKa of the acid (pKa = 4.76). In acidic environments, like NWS, many organic acids are in non-dissociated forms and can penetrate the cell membrane, accumulating within the cytoplasm and causing loss of viability and cell death^24^. Accordingly, the acetic acid produced by NWS mixed cultures is mainly in the non-dissociated at pH < 4.5 and it can penetrate the cytoplasm producing deleterious effects on the cells. Ethanol was detected at low concentrations (data not shown).

Presumptive thermophilic LAB were estimated in three different conditions, namely MRS (pH 5.4) medium anaerobically (for lactobacilli enumeration); M17 medium supplemented with sterile skimmed whey (M17-SSW) aerobically (for streptococci enumeration), and M17-SSW medium anaerobically (Fig. 1B–F). All the samples showed presumptive lactobacilli counts ≥ than 7.5 Log_10_ CFU/mL (Fig. 1B), while cocci-shaped populations enumerated both aerobically and anaerobically on M17-SSW medium ranged from 6.65 ± 0.23 to 8.37 ± 0.17 Log_10_ CFU/mL (Fig. 1C,D). The samples C2, C7, and C10 showed the lowest streptococci counts as enumerated on M17-SSW medium aerobically (*p* < 0.05). Less remarkable differences were detected in the plate counts on M17-SSW medium incubated under anaerobic conditions (Fig. 1D).

Mesophilic yeasts were detected in all samples, with C1 showing the highest counts (*p* < 0.05) (Fig. 1E). Selective enumeration on YPLA medium at 42 °C showed that thermotolerant and lactose-fermenting species represent the dominant yeast fraction (Fig. 1F). These results agreed with data previously collected from other PR-NWS samples^25,26^. The lack of detectable level of ethanol in all NWS suggest that yeasts either preferably consume lactate or engage a respiratory sugar catabolism.

### Cultivable bacterial fraction characterization

After several attempts, we successfully isolated 57 Gram positive bacterial axenic cultures; catalase reaction was negative for all the isolates, except for T2013. We observed a decline in cultivability of isolates in all three culture conditions. Fifty-three percentage of colonies either did not grow after the first purification step or died during the subsequent rounds of purification by streaking on the same isolation medium. The cultures most recalcitrant to isolation were those from M17-SSW medium incubated in anaerobiosis at 42 °C. This apparently disagrees with the assumption that media that mimic environmental conditions should increase and diversify the number of cultivable bacteria.

16S-ARDRA with the selected restriction enzyme *Mse*I discriminated all the species considered, except for *L. delbrueckii* subsp. *bulgaricus* and *L. delbrueckii* subsp. *lactis* (Supplementary Table S2). Differentiation between *L. delbrueckii* subsp. *bulgaricus* and *L. delbrueckii* subsp. *lactis* was successfully obtained with the enzyme *Eco*RI^27^. Analyzing the electrophoretic profiles obtained with *Mse*I, out of 57 NWS isolates 56 were classified into three species, namely*, L. helveticus* (33.90%), *L. delbreueckii* (22.03%), and *St. thermophilus* (38.98%). Culture conditions affected the species recovery, with MRS (pH 5.4) selecting *L. helveticus* and M17-SSW under anaerobiosis *L. delbrueckii*, respectively. The medium M17-SSW under aerobic conditions was confirmed to be selective for *St. thermophilus*^28^. All the *L. delbrueckii* isolates resulted to be assigned to subsp. *lactis*, according to *EcoR*I restriction pattern (Supplementary Table S2).

**Figure 2:**
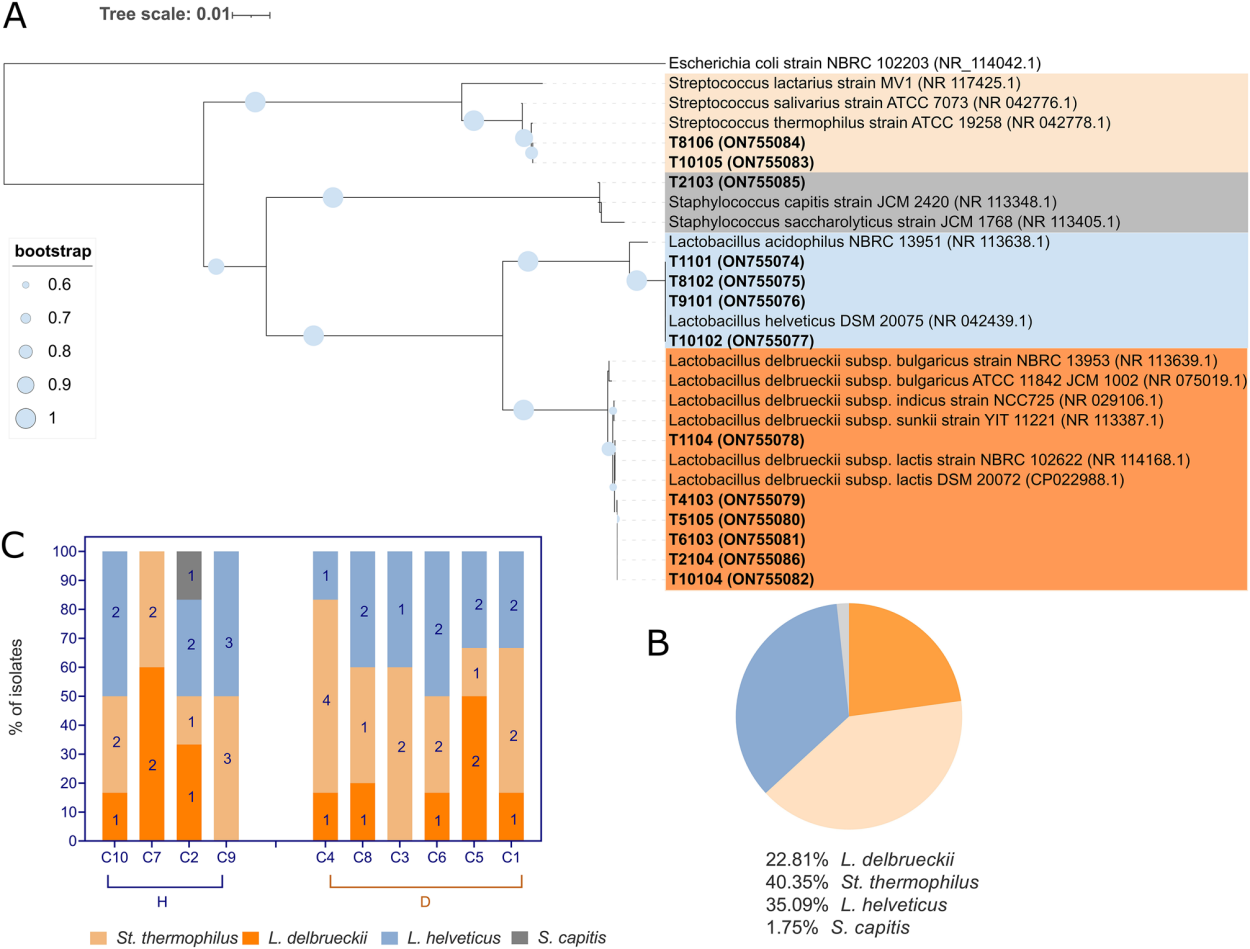
Characterization of cultivable microbial fraction of NWS. (**A**). Evolutionary relationships of thirteen NWS microbial isolates (in bold) inferred using the Neighbor-Joining method. The percentages of replicate trees (values higher than 60%) in which the associated taxa clustered together in the bootstrap test (1000 replicates) are shown as circles next to the branches. The evolutionary distances were computed using the Kimura 2-parameter method rate variation among sites was modeled with a gamma distribution (shape parameter = 1). The analysis involved 27 nucleotide sequences and *E. coli* 16S rRNA gene partial sequence (NR_114042.1) was used as outgroup. (**B**) Pie-chart depicting microbial species frequencies. (**C**) Species distribution in each NWS sample. Numbers on the column represent biotypes scored by UPGMA analysis of (GTG)_5_ rep-PCR fingerprinting data. H and D agree with NWS types obtained through 16S rRNA metabarcoding analysis. (**A**) was visualized and edited with iTOL (available at https://itol.embl.de/), while (**B**,**C**) with GraphPad Prism v.8.00 software (San Diego, CA, USA, https://www.graphpad.com/).

A total of 27 high-quality sequences obtained from 13 bacterial isolates representative of the previously established restriction patterns and 14 reference strains retrieved from GenBank RefSeq database was used to infer phylogenetic positions of bacterial cultures (Fig. 2A). A total of three major clades were represented, including the genera *Streptococcus*, *Lactobacillus*, and *Staphylococcus*, respectively. One strain, T2103, was grouped with *Staphylococcus capitis* ATCC 19258^T^, sharing 100% similarity. Two strains, T10105 and T8106, were grouped with *St. thermophilus* ATCC 19258^T^, sharing 100% similarity among them and 99.93% similarity with the reference strain. The remaining 10 isolates were included into the *Lactobacillus* clade. Within this clade branches representing species differentiation were supported by high bootstrap values for all the species considered, except for *L. delbrueckii*. Five bacterial isolates formed a monophyletic cluster with *L. helveticus* DSM 20075^T^. The branching was supported by 100% bootstrapping value. The discrimination among *L. delbrueckii* subspecies was poorly supported as expected from the high similarity in 16S rRNA gene sequence within species^29^. Five strains were grouped within the *L. delbrueckii* clade (100% bootstrapping), nearer to *L. delbrueckii* subsp. *lactis* than to *L. delbrueckii* subsp. *bulgaricus*. No isolates belonging to *L. fermentum* were found.

To assess the extent of intra-species diversity, we genotyped the isolates using the micro-satellite region (GTG)_5_ in rep-PCR. After UPGMA clustering analysis, a total of 34 genotypes was found with a reproducibility cut-off of 88% (Supplementary Fig. S1). From 34 genotypes 20 were singletons and accounted for the 58.82% of inter-individual variability. We divided *L. delbrueckii* into 4 subclusters and 3 singletons, *L. helveticus* into 5 subclusters and 6 singletons, and *St. thermophilus* into 5 subclusters and 10 singletons. More than one biotype was present for every species within each sample. Simpson (1-*D*) a-diversity index obtained for the total population of 34 genotypes was 0.68. Based on the overall results, the cultivable fraction was composed by *St. thermophilus* as dominant species, followed by *L. helveticus* (Fig. 2B). Species distribution was variable across the samples, with *L. helveticus* accounting for the 50% of isolates in only three samples (C6, C9, and C10, respectively) (Fig. 2C). *Staphylococcus capitis* was found only in C7 and appeared an occasional contaminant maybe related to low milk quality.

### Bacterial community profiling

Problems in LAB cultivability suggested us to attempt 16S rRNA metabarcoding as alternative approach for profiling bacterial community of NWS samples. A total of 4,325,460 raw paired-end sequences were obtained from the 10 NWS samples considered in this study. After denoising, 2,162,730 paired end reads were retained with an average of 40,210.52 reads per sample (range 23,726.0 to 66,119.0). A total of 55 ASVs having more than 4 reads were identified, of which 45 were further selected at 0.001% of read frequency to eliminate the underestimated ASVs. The rarefaction curves reached the plateau for all NWS samples, suggesting that a complete coverage of the NWS bacterial community had been reached through the sequencing depth used (Supplementary Fig. S2).

**Figure 3:**
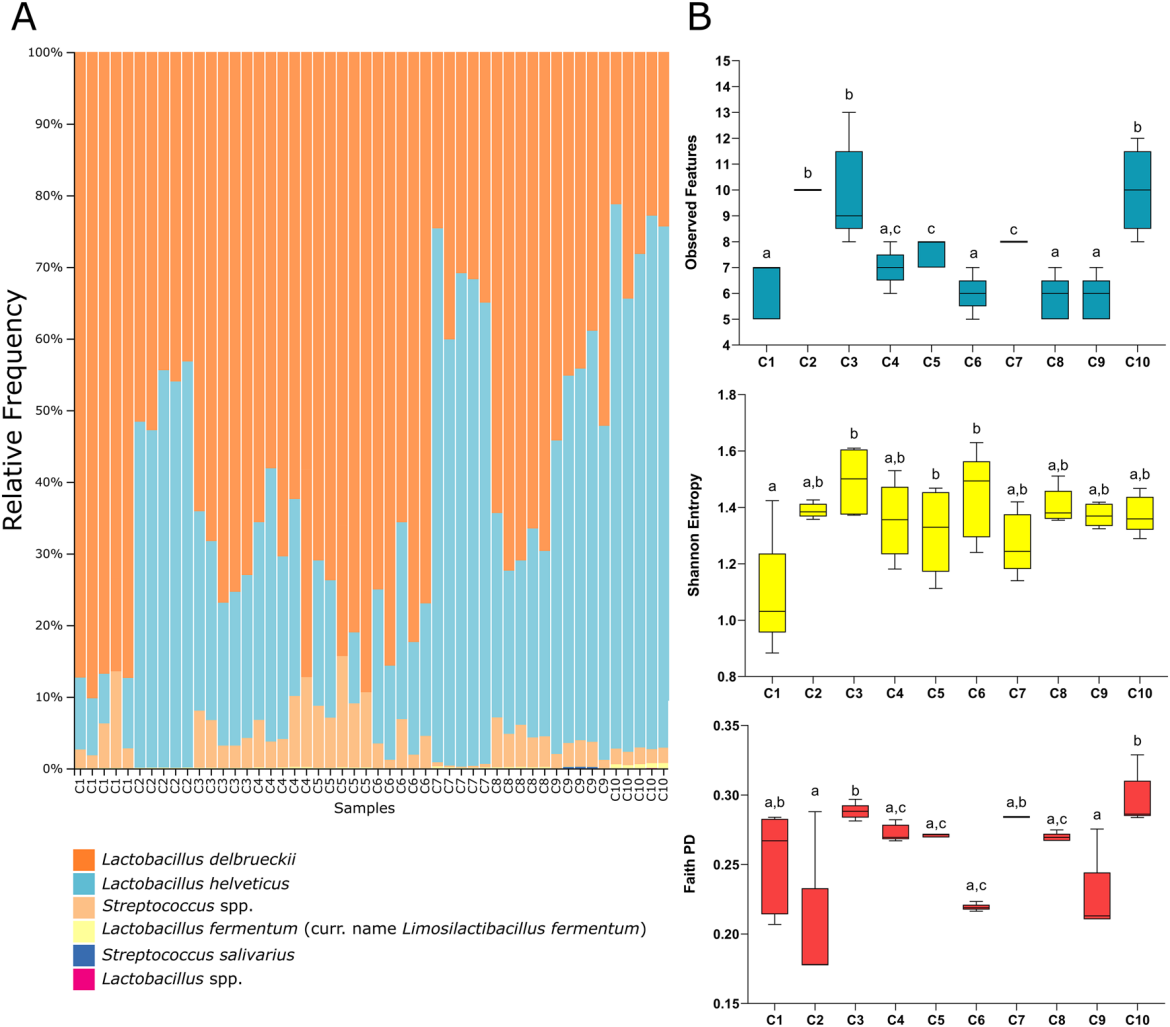
Relative abundance and alpha diversity analysis of NWS samples. (**A**) Cumulative bar chart representing the relative abundance on the vertical axis and the NWS samples (treatments; *n* = 50) on horizontal axis. Only taxa contributing to more than 0.1% of the total abundance in at least one sample are shown. (**B**) Observed features, Shannon index, and Faith’s PD index. Plotted with GraphPad Prism v.8.00 software (San Diego, CA, USA, https://www.graphpad.com/).

The ASVs passing the quality control were aligned and subjected to taxonomic profiling. Based on Silva database annotation, 6 taxa were observed and most 16S rRNA gene sequences (95.7%) were classified at species level. Four species were identified, namely *L. helveticus*, *L. delbrueckii*, *Streptococcus* spp., and *L. fermentum* (Fig. 3A). Manual BLAST search revealed that the remaining two taxa were ascribed to *L. delbrueckii* and *S. salivarius* subsp. *thermophilus*.

To evaluate the taxa abundance, alpha diversity metrics (richness, Shannon index, and Faith’s PD) were calculated within each sample replicates (*n* = 5), regarded as a community (Fig. 3B). The richness index was significantly affected by the dairy farm (H = 41.15, *p* < 0.001), with the highest bacterial richness in samples C2, C3, and C10, whilst the lowest richness in C1, C6, C8, and C9, respectively (Fig. 3B). The Shannon index accounts for both richness and evenness, with higher values indicating more diverse and uniformly distributed ASVs. The site of production did not significantly influence the Shannon index (H = 15.15; *p* > 0.05). Sample C1 significantly differed from samples C3, C5 and C6 in Shannon index. Although C1 and C6 had similar low richness, they differed in Shannon index, suggesting that C6 had a lower number of more highly distributed observed features compared to C1 (Fig. 3B). Faith’s PD, which additionally incorporates the phylogenetic distance of the ASVs, was strongly affected by cheese factory (H = 33.75; *p* < 0.001) with NWS C2, C6, and C9 showing the lowest phylogenetic diversity (Fig. 3B).

**Figure 4:**
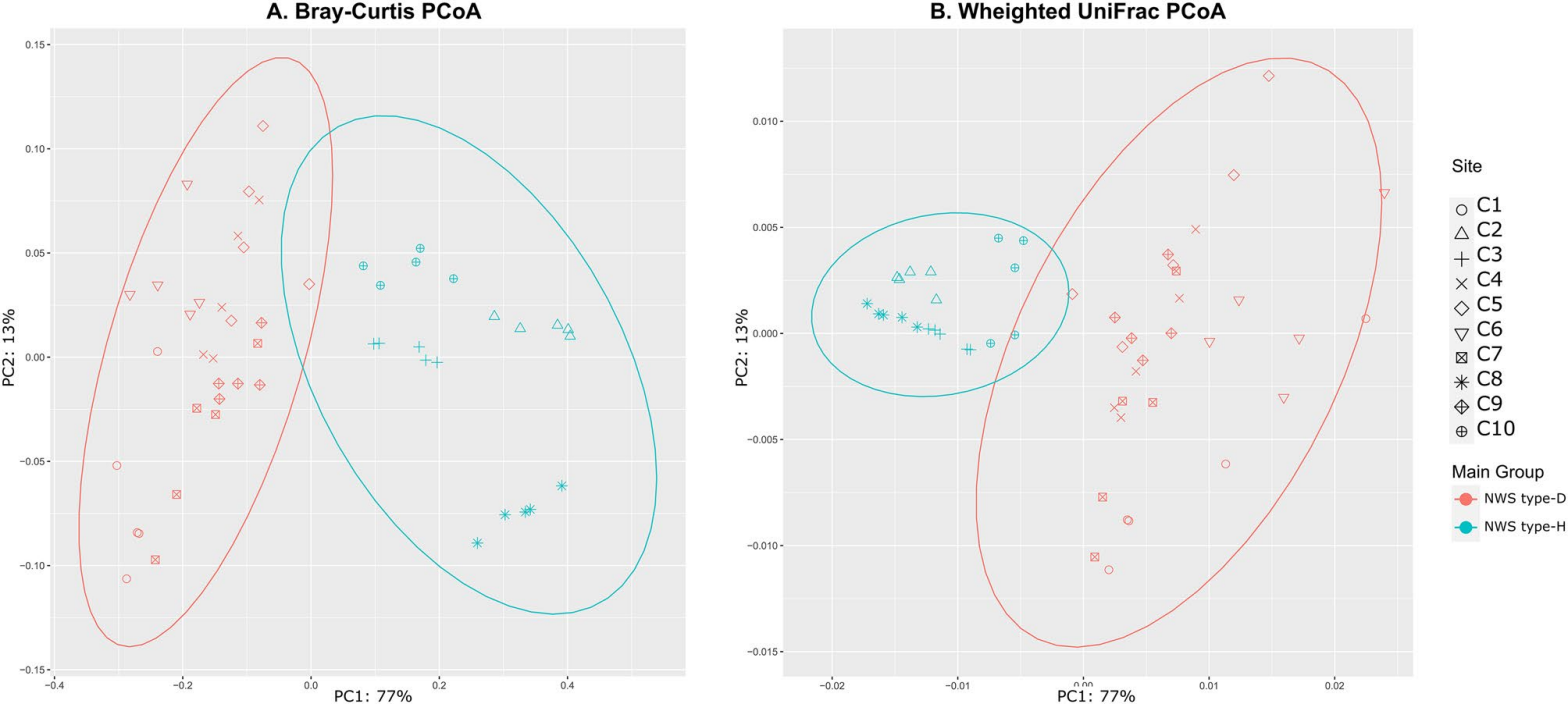
Beta diversity plots of NWS samples based on Principal coordinates analysis (PCoA) of (**A**) Bray–Curtis and (**B**) weighted Unifrac distance. Points represent samples, while color indicates dairy farms. The percentage reported on axes represent the amount of total variance depicted by each of them. (**A**,**B**) were plotted with R v.4.1.1, while the ellipses were calculated and drawn with 0.95 of confidence level using ggplot v.2 3.3.5 (stat_ellipse).

Beta diversity provides an overview of the similarities in the bacterial communities between dairy farms. Principal coordinate analysis (PCoA) plots of the Bray–Curtis and weighted UniFrac indexes showed two significant groups of samples, termed type-H (C2, C7, C9, and C10) and type-D (samples C1, C3, C4, C5, C6, and C8) (Fig. 4A,B, respectively). No significant differences were found among cheese factories in Jaccard and unweighted UniFrac distances (data not shown). Since the weighted UniFrac distance accounts for ASVs abundance and unweighted UniFrac distance for the presence/absence of ASVs, we supposed that the main source of differences among NWS samples was the individual microbial abundance rather than species composition. PERMANOVA analysis was applied to the distance dataset in order to find significant differences among the treatments. The test revealed that cheese factory strongly affects microbial population diversity among the samples (Bray–Curtis PERMANOVA: pseudo-F = 80.53; *p*-value = 0.001).

### Bacterial signature detection

**Figure 5:**
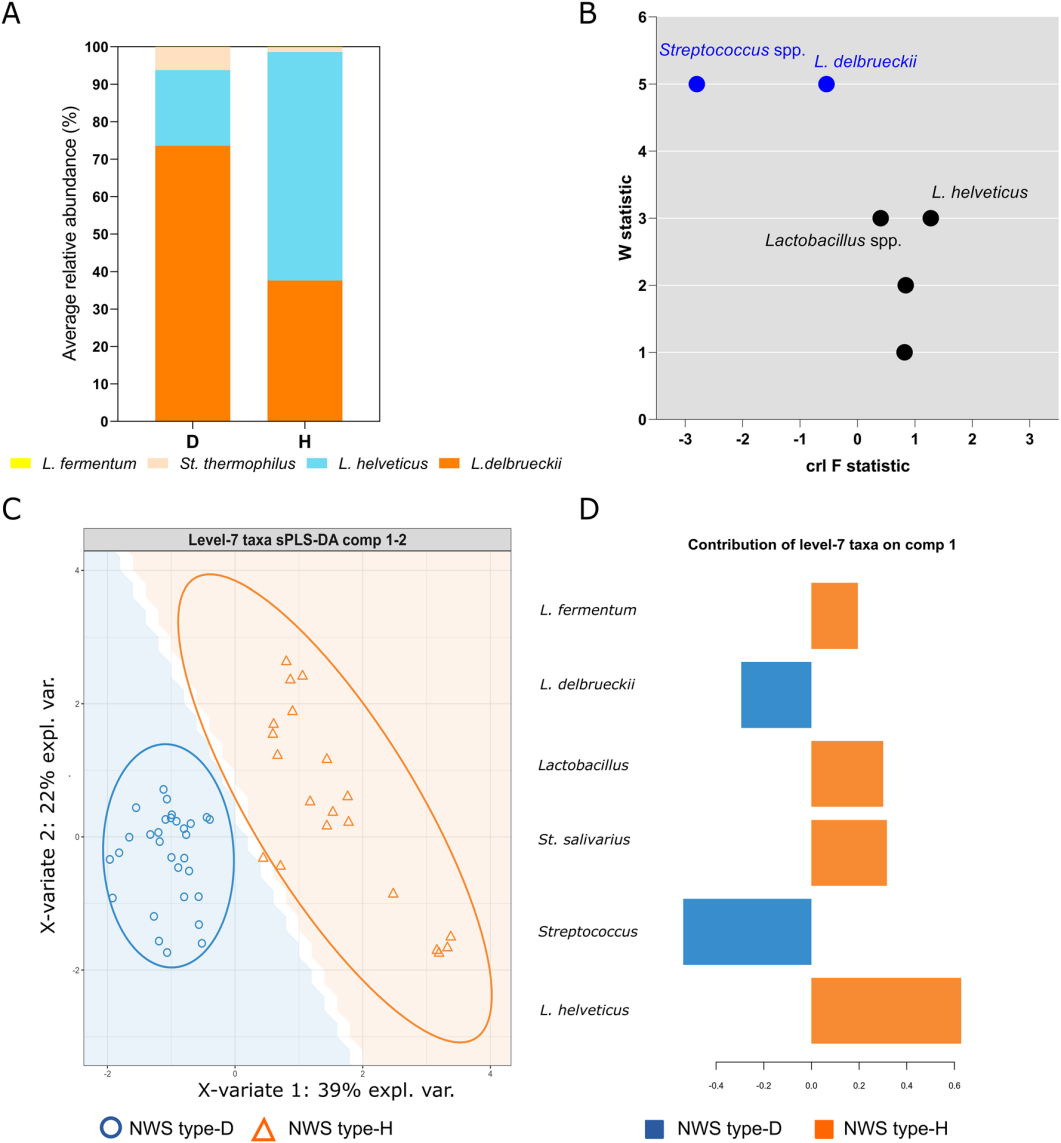
(**A**) Species level composition of NWS type-D and type-H. The average relative abundances of each bacterial taxon and clusters are reported on the vertical and horizontal axis, respectively. (**B**) ANCOM differential abundance volcano plot. (**C**) sPLS-DA of the NWS samples (*n* = 50) showing discrimination between samples H (blue circles) and D (orange triangles). (**D**) Loading plot shows the discriminant power of species in explaining differences between groups on component PLS1. The direction of the bars (left or right) relates to the direction of the loadings in panel A. The higher the absolute value, the bigger is the discriminative power. Orange and blue bars indicate a higher abundance in D or H group, respectively. (**A**) was plotted with GraphPad Prism v.8.00 software (San Diego, CA, USA, https://www.graphpad.com/), while (**B**–**D**) were built using mixOmics R package v.6.16.0 (https://CRAN.R-project.org/package=mixOmics).

Considering only the ASVs whose relative abundance in each sample was greater than 0.001%, *Firmicutes* resulted the dominant phylum, that describes more than 99% of the bacterial microbiota in all the samples. *Lactobacillus helveticus* and *L. delbrueckii* were the dominant taxa, followed by *Streptoccoccus* spp. and *L. fermentum*. We also observed that *L. delbrueckii* and *Streptoccoccus* spp. represent 79.7% of the total average relative abundance in NWS type-D, while *L. helveticus* had an average relative abundance of 60.9% in NWS type-H. *L. fermentum* was detected only in NWS cluster H at low relative abundance (0.24%) (Fig. 5A).

To determine significant compositional differences across groups, we used the statistical framework ANCOM^30^, which infers absolute abundance from relative abundance data and can detect which taxa are differently abundant across groups. ANCOM analysis identified *Streptococcus* spp. (w = 5) and *L. delbrueckii* (w = 5) as different between groups. These species were more abundant in cluster D than in cluster H (Fig. 5B).

To detect specific ASVs which contribute to separate NWS samples into type-D and type-H, sPLS-DA was performed. sPLS-DA is a multivariate method performed on the clr-transformed microbiome data to identify microbial drivers or biomarkers discriminating samples groups. sPLS-DA plot showed that PLS1 and PLS2 explain 39 and 22% of the variation in NWS microbiota composition, respectively, and effectively separate cluster H from cluster D (Fig. 5C). To further identify the specific taxa that were predominant as the biomarkers between the groups, the contribution to PLS1 was calculated. *Streptococcus* spp. was the most significant taxon in NWS type-D followed by *L. delbrueckii*, while *L. helveticus* characterized NWS type-H (Fig. 5D).

### Differential analysis of predicted functional content

**Figure 6:**
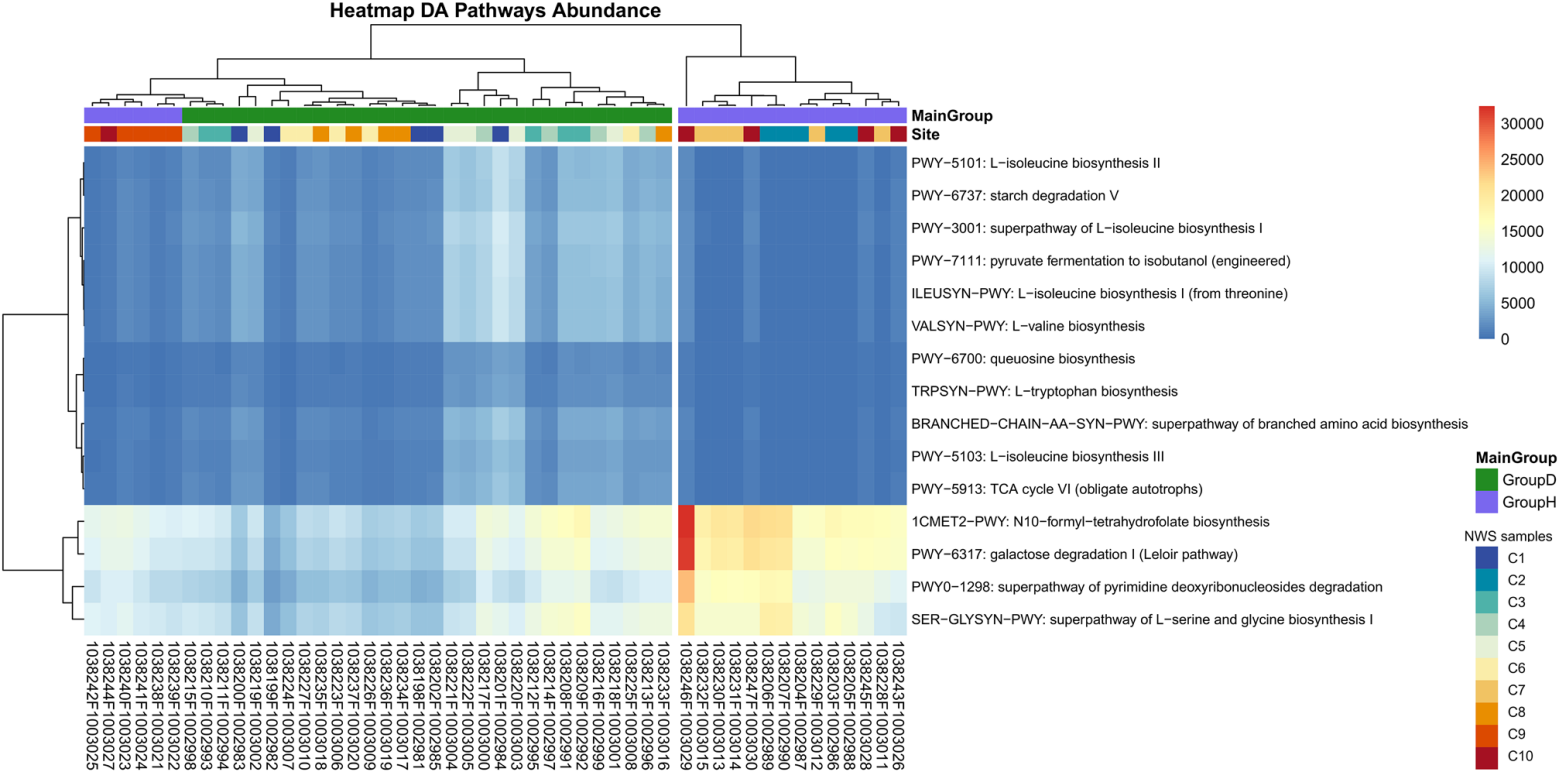
Functional heatmap. Hierarchical clustering heatmap visualized with pheatmap R package v.1.0.12 shows samples in columns and the 15 most characterizing MetaCyc pathways in rows. The PICRUSt2-predicted abundance levels are represented by the background color, where blue means low and red means high abundance. The main experimental conditions are shown in green and violet, representing NWS type-D and NWS type-H, respectively.

To provide a preliminary functional insight for the taxonomic profiles observed in our study, we used the PICRUSt2 software^31^ and we performed the differential abundance analysis with three independent tools, such as ALDEx21.26.0^32^, ANCOM-BC 1.4.0^33^, and MaAsLin2^34^. The PICRUSt2 analysis predicted the presence of 1345 functional gene orthologs, 589 enzymes and 118 metabolic pathways across all NWS samples. Fifty-three metabolic pathways reported in the MetaCyc database were significant as demonstrated by ALDEx2 1, ANCOM-BC, and MaAsLin2 analyses, respectively. The heat map of 15-top pathways showed that NWS type-H and D differed from each other in some metabolic functions (Fig. 6). In detail, samples were assigned to 4 clusters based on the composition of predicted metabolic pathways. One included almost all samples H, except for C9 and one replicate of sample C10. Four pathways were enriched in cluster H; among them, we found the galactose degradation I pathway and the N10-formyl-tetrahydrofolate biosynthesis, involved in purine biosynthesis. NWS type-D appeared enriched in pathways related to aromatic and branched chain amino acid (BCAA) biosynthesis (Fig. 6).

### Spearman correlation analysis

**Figure 7:**
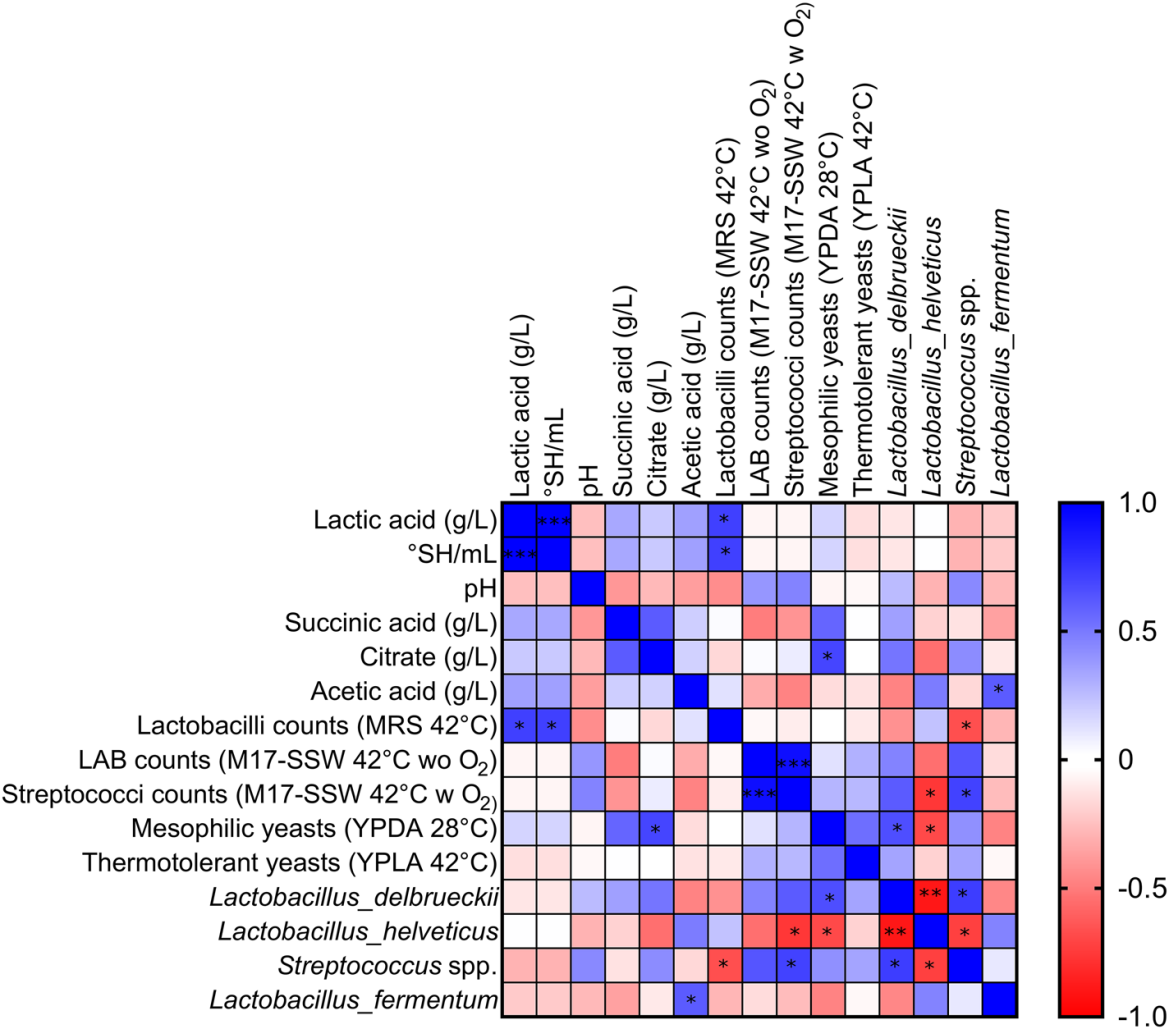
Spearman’s correlation analysis of relative abundance of 4 selected ASVs with plate counts, physicochemical parameters, and organic acid concentrations. Blue to red scale denote positive to negative associations. Spearman’s correlations were employed in agreement with data distribution and verified by Kruskal–Wallis test. *p < 0.05, **p < 0.01, ***p < 0.0001 following the Spearman’s correlations. Plotted with GraphPad Prism v.8.00 software (San Diego, CA, USA, https://www.graphpad.com/).

Spearman correlation analysis assessed the relationships between taxa, plate counts, and physicochemical/technological outcomes (Fig. 7). As expected, lactic acid concentration showed a strong positive correlation with titratable acidity (*r* = 1.0; *p* = 5.511 × 10^–7^), in agreement with the high amount of this acid detected in NWS. Lactobacilli counts were positively correlated to lactate concentration and titratable acidity (*r* = 0.721; *p* = 0.023) and negatively correlated with the abundance of *Streptococcus* spp. (*r* = 0.661; *p* = 0.044). This negative relationship agreed with previous sPLS-DA analysis. As expected, abundance of *L. delbrueckii* was negatively correlate with abundance of *L. helveticus* (*r* = 0.981; *p* = 0.044), whilst relative abundance of *Streptococcus* spp. negatively correlated with abundance of *L. helveticus* (*r* = − 0.721; *p* = 0.023) and positively correlated with counts of presumptive streptococci population (*r* = 0.709; *p* = 0.027) (Fig. 7). This last result further confirms that M17-SSW medium under aerobiosis represents the appropriate cultivation condition for detecting *St. thermophilus*, as previously reported^28^.

Remarkably, mesophilic yeast population was positively related to the abundance of *L. delbrueckii* (*r* = 0.661; *p* = 0.044) and negatively related to *L. helveticus* (*r* = − 0.685; *p* = 0.035). This result suggests that NWS type-D could be more prone to yeast contamination than NWS type-H. Furthermore, mesophilic yeasts were positively correlate with citrate concentration (*r* = 0.709; *p* = 0.031) (Fig. 7). Yeasts inhabiting NWS are generally Krebs-positive species suitable to use both lactate and Krebs cycle intermediates as carbon and energy sources^35^. Although not statistically significant (*r* = 0.697; *p* = 0.067), there was a positive correlation between abundance of *L. fermentum* and acetic acid concentration, in agreement with the heterofermentative catabolism of this species^22^.

### Acidification assays in milk

**Figure 8:**
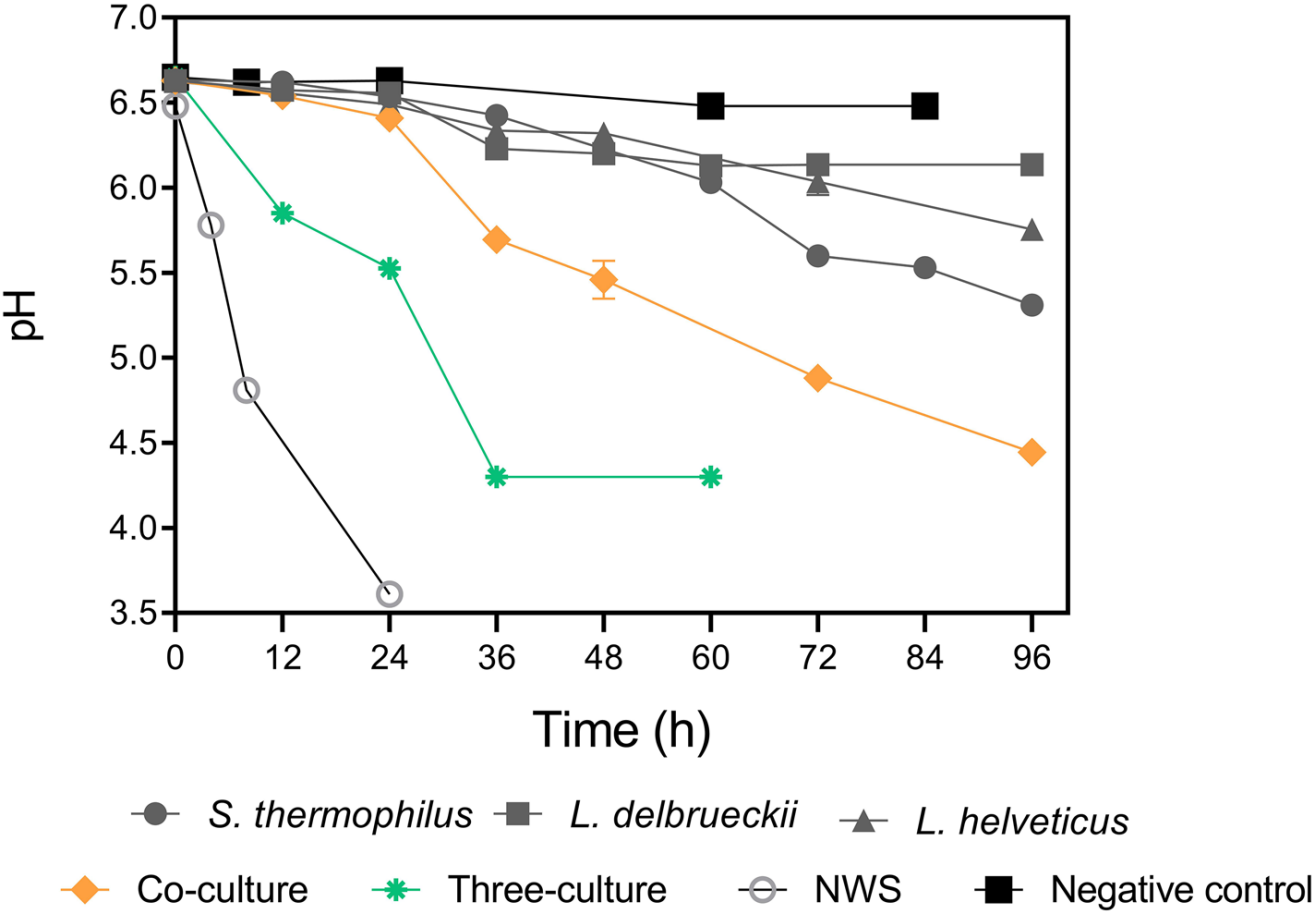
Acidification curves of *St. thermophilus*, *L. helveticus* and *L. delbrueckii* susp. *lactis* axenic cultures isolated from NWS. Tester strains were inoculated in milk as monocultures (grey), coculture (*St. thermophilus* RBC06 x. *L. delbrueckii* susp *lactis* CBB09) (orange), and triculture (*St. thermophilus* RBC06 x. *L. delbrueckii* susp *lactis* CBB09 × *L. helveticus* RBB04) (green). Milk inoculated with fresh NWS (light grey) and uninoculated milk (black) were used as positive and negative controls, respectively. Values are mean of at least three replicates. Bars when visible represent standard deviation values. Plotted with GraphPad Prism v.8.00 software (San Diego, CA, USA, https://www.graphpad.com/).

Difficulty in axenic culture cultivation and co-occurrence of *St. thermophilus* and *L. delbrueckii* in NWS type-D suggested that mutualistic interactions could take place between NWS species and that these bacteria can grow better together than alone. To test this hypothesis, we assessed the milk acidification performance of three randomly selected tester strains representative of species *L. delbrueckii*, *L. helveticus* and *St. thermophilus*, respectively. All tester strains exhibited slow acidification curves as monocultures, with *L. delbrueckii* CBB09 showing the slowest acidification trend (*p* < 0.05) (Fig. 8). When *St. thermophilus* was cocultured with *L. delbrueckii*, trend in acidification significantly increased (*p* < 0.05). Tricultures of *St. thermophilus* RBC06, *L. delbrueckii* CBB09, and *L. helveticus* RBB04 resulted in faster acidification trend compared to both monocultures and cocultures (Fig. 8). Significantly, NWS sample outcompeted both tricultures and cocultures in pH decrease.

## Discussion

Undefined cultures are complex bacterial communities where there are usually multiple strains per species and only a few species being dominant^36^. Here, we proposed the existence of two main NWS community types in PR cheesemaking, named NWS type-H and NWS type-D. Notably, the distinctive feature that characterizes NWS type-H and NWS type-D is the dominance of NWS microbiota by *L. helveticus* and *L. delbrueckii*/*St. thermophilus*, respectively. Intriguingly, co-occurrence of *L. delbrueckii* and *St. thermophilus* in NWS type-D has been also documented in a Swiss hard cheese starter, where these species form a stable community and engage mutualistic interactions by metabolite exchanges^37^. Like in yogurt cultures, they metabolically complement each other: *L. delbrueckii* is as protease-competent species which provides amino-acids to the poorly proteolytic species *St. thermophilus*^38^, meanwhile *St. thermophilus* possesses metabolic pathways for folate, lactate, and formate production^39^. In our case the higher relative abundance of *St. thermophilus* in samples dominated by *L. delbrueckii* supports that similar cross-feeding relationships could exist in PR NWS too. The observed reduced cultivability of axenic cultures obtained from PR NWS samples supported this hypothesis. Application of metagenomic approaches and reconstruction of metagenome-assembled genomes (MAG) from PR NWS will better elucidate this metabolic complementation.

Differently from the Swiss hard cheese starter^37^, in PR NWS communities *L. helveticus* plays a key role other than *L. delbrueckii* and *St. thermophilus*. Coexistence of these species makes PR NWS very similar to the natural starter used for Swiss Gruyère-Type cheese^40^. Our results showed a differential presence of *L. helveticus* in NWS type-H compared to NWS type-D. This species was inversely related to *St. thermophilus*, but no samples were found with *L. helveticus* as being the only species present. *L. helveticus* is generally considered positive in dairy fermentation as this bacterium overcomes the multiple amino acid auxotrophies through a large set of proteases and peptidases which breakdown proteins and peptides into amino acids^41^. The proteolytic system of *L. helveticus* has been associated with important dairy traits, such as fast cheese ripening, enhanced flavor development, and reduced bitterness^42^. However, the lack of PR NWS samples with only *L. helveticus* as dominant species could be positive as overdominance of *L. helveticus* reduces rheological properties and cheese elasticity due to an excessive proteolysis and carbon dioxide production^40,43^. The correct balance between the LAB species in dairy ecosystems supports that the inter-species interactions are responsible for both the development of the expected organoleptic complexity and texture, and the resilience toward stress conditions and colonization by spoilage microbes^8^. Compared to previous studies^12^, no *L. fermentum* was detected except for sample C10. In Trentingrana cheese NWS more species were detected other than *L. helveticus*, including *Lacticaseibacillus paracasei*, *Lactiplanctibacillus plantarum*, and *L. fermentum*^44^.

Among spoilage microbes, yeasts can utilize lactose and lactate reducing NWS acidifying performance. Among yeasts isolated from PR NWS, only *Kluyveromyces marxianus* breakdowns lactose, but all the other species, such as *Torulaspora delbrueckii*, *Wickerhamiella pararugosa*, and *Saccharomyces cerevisiae*, are galactose-fermenting yeasts^26^. The inverse relationship between yeast contamination and *L. helveticus* could depend on the ability of *L. helveticus* to ferment both glucose and galactose, leaving no sugar moieties available for yeast respiration. In contrast, *L. delbrueckii* is generally described as Gal^-^ species^45^, while in metadatabase BacDive (http://bacdive.dsmz.de) *St. thermophilus* has been reported as variable in galactose fermentation. A lower ability to consume galactose in PR NWS type-D could explain the higher yeast contamination observed in these samples. In the attempt to provide a preliminary functional insight for the taxonomic profiles observed in our study, we used PICRUSt2 software and three different algorithms for the differential abundance analysis of predicted pathways. The results suggest that bacterial pathways involved in purine biosynthesis and galactose catabolism were significantly represented in NWS type-H, while branched chain and aromatic amino acids biosynthetic pathways were overrepresented in NWS type-D. Tryptophan and BCAA are important precursors of flavor compounds^46^ and could suggest that NWS type-D could differentially impact the quality of PR cheese compared to NWS type-H both in terms of residual amount of galactose and flavor compound precursors. In accordance with our data, Santarelli et al.^47^ divided PR cheeses during moulding phase in two groups, one characterized by high lactate level and dominated by *L. helveticus* and the other one with a low lactate level and a high level of *St. thermophilus*.

Remarkably, uncultivability of individual single colony strains did not allow us to collect enough isolates to depict a complete picture of the intra-species diversity in PR NWS samples. Poorly cultivability also determined little consistency of culture-dependent data with respect to the 16S rRNA metabarcoding profiles. Reasons for this uncultivability could be several, from phage infection to the lack of metabolic complementation that occurs in undefined starter community. Although being partial and preliminary, genotyping data collected from the survived single colonies showed a high degree of genetic heterogeneity at strain level rather than at species level, with more than one biotype per species within each sample and different biotypes among samples. Similar results have been reported in other undefined starter communities^6,37,48^. The intraspecies diversity is thought to be responsible for resilience against environmental uncertainty^49^ and is linked to functionally adaptive traits encoded by genomic islands and mobile genetic elements^50^. Bacteriophages could have an additional role to regulate population diversity through density-dependent predation^51^. The ‘Kill-the-winner’ model predicts that phage predation ensures diversity by suppression of the more abundant strains^52^. Unfortunately, 16S rRNA metabarcoding approach adopted in this study neither characterized phages nor determined CRISPR arrays variability in *L. helveticus*, *St. thermophilus* and *L. delbrueckii* MAGs. However, undefined bacterial communities similar to PR NWS, like Trentingrana NWS, were proved to be contaminated by lysogenic phages^44^. Somerville et al.^37^ also demonstrated that genomic differences among isolates from Swiss hard cheese starter are mainly due to CRISPR spacers. All these findings support that phage-bacteria interactions shape bacterial diversity in undefined NWS.

Our growth experiments showed that monocultures of *St. thermophilus*, *L. bulgaricus*, and *L. helveticus* isolated from PR NWS have poor acidifying ability, with *L. delbrueckii* subsp. *lactis* exhibiting the worse performance followed by *St. thermophilus*. This agrees with previous observations in yogurt where monocultures of *St. thermophilus* or *L. bulgaricus* subsp. *bulgaricus* grow slowly when milk was not supplemented with amino acids and formate, respectively^39^. In our experiment *L. helveticus* also grew slowly in milk. This disagrees with the general assumption that *L. helveticus* is a fast-acidifying species^42^. *L. helveticus* strains with a slowly milk-coagulating phenotype have been previously isolated from undefined starters^53^ and Mongolian fermented milk^54^. This phenotype is linked to the loss of plasmids harboring *prt* genes^53^, loss of aminopeptidases-encoding genes^55^, and deficiency in purine biosynthesis^56^. Gene decay and genome reduction have been frequently documented in microbes inhabiting nutritional rich environments^57^. According to the Black Queen hypothesis^58^, protease-negative strains can invade the population and dominate over the protease-positive strains^59^. The progressive increase in milk acidification rate of co-cultures and tri-cultures supports that *L. helveticus* also could benefit from cross-feeding relationships with *St. thermophilus* and *L. bulgaricus* subsp. *lactis*. These results agree with previous observation that individual strains of NWS thermophilic lactobacilli grown in whey can benefit from the presence of cell-free supernatant fluids from whey cultures of other strains^60^. Massive milk acidification tests with a wider number of strains *per* species will confirm these preliminary observations and will help to decipher mechanisms underpinning these metabolic interactions.

In conclusion, the present study demonstrated that PR NWS are bacterial communities that can be clustered in two types based on differences in species abundance and that they are shaped by complex relationships at inter and intra-species level. Further studies based on metagenomic HST of a broader assortment of NWS samples will be of interest to confirm whether metabolite complementation and phage infection are the driving forces of these interactions. The functional and genomic characterization of PR NWS strains isolated in this study will also led light on intra-species distribution of relevant ecological and technological phenotypes, such as proteolytic ability, galactose fermentation, and folate and purine biosynthesis.

## Materials and methods

### Reference strains and culture conditions

Type strains and tester strains used in this study were *St. thermophilus* DSM 2061^T^ and RBC06; *L. helveticus* DSM 20075^T^ and LBB04; *L. delbrueckii* spp. *lactis* DSM 20072^T^ and CBB09; *Lactobacillus delbrueckii* subsp. *delbrueckii* DSM 20074^T^ and *L. fermentum* DSM 20052^T^. All the type strains were from DSMZ collection and cultivated according to DSMZ growth conditions. Tester strains *St. thermophilus* RBC06, *L. helveticus* LBB04, and *L delbrueckii* subsp. *lactis* CBB09 were previously isolated from PR NWS and 16S rRNA gene sequenced (GenBank accession numbers OM891849, ON936798, and ON936797, respectively).

### Sampling and physicochemical analyses

Ten NWS samples were collected in the PDO area of the PR cheese. All the samples were collected in October 2021 to minimize seasonal variations. Geographical localization and details on dairy farm management system were reported in Supplementary Table S3, while temperature curves used for NWS production in Table S4, respectively. When necessary, samples were collected from multiple fermentation units in each dairy farm and then mixed. Samples were kept immediately at 4 °C and transported in laboratory for further analyses. pH was measured directly using a pH meter (Crison Instruments, Barcelona, Spain), without dilution. Titratable acidity was determined in 50 mL of NWS using the Soxhlet–Henkel method with 0.25 N NaOH and recorded in Soxhlet–Henkel degrees (°SH/50 mL)^26^. Acidification rate of NWS samples was assessed as previously reported^61^ and expressed as Δ°SH/50 mL. Lactic, succinic, acetic, and citric acids were enzymatically determined, according to manufacturer’s instructions (Megazyme, Wicklow, Ireland).

### Microbial enumeration and viable fraction characterization

Microbiological analyses were carried out by ten-fold diluting the samples in physiological water (9 g/L NaCl) and spread them on: de Man-Rogosa-Sharp (MRS; Oxoid, Milan, Italy) medium (brought to pH 5.4 with 1 N HCl) for the enumeration of the presumptive lactobacilli; M17 medium^62^ supplemented with SSW (Morga AG, Ebnat-Kappel, Switzerland) (M17-SSW) at the final concentration of 7% v/v for the numeration of presumptive cocci LAB; YPDA medium for the generalist yeast enumeration; and YPLA for the enumeration of lactose fermenting yeasts. The SSW was prepared according to Fornasari et al.^28^. MRS and M17 media were supplemented with cycloheximide at the final concentration of 20 mg/L, while YPDA and YPLA media with chloramphenicol at the final concentration of 10 mg/L. MRS plates were incubated at 42 °C for 48 h under anaerobiosis (Oxoid, Milan, Italy); M17-SSW plates at 42 °C for 72 h both under aerobic and anaerobic conditions; YPDA and YPLA plates at 28 °C and 42 °C for 48 h, respectively. For anaerobic conditions, Oxoid AnaeroGen system (Thermo Fisher Scientific, Waltham, MA, USA) was used. Viable cell counts were recorded as number of colony forming units (CFU)/mL recovered from plates with colonies ranging from 20 to 200 and expressed as Log_10_ CFU/mL means of at least three replicates.

Bacterial colonies were submitted to at least two rounds of streaking and then characterized for micromorphology, Gram staining, and catalase test. All the clones collected in this wok were conserved ex situ at − 80 °C in liquid medium supplemented with 25% (v/v) glycerol. Bacterial cultures were submitted to DNA extraction as previously reported^63^ and preliminarily distinguished into clusters by Amplified 16S Ribosomal DNA Restriction Analysis (16S-ARDRA) using the diagnostic endonuclease *Mse*I (Thermo Scientific, Waltham, MA, USA). This endonuclease was selected based on in silico analysis of the 16S rRNA gene sequences of the most frequently recognized LAB species in NWS carried out on with Snapgene software (www.snapgene.com). Briefly 16S rRNA gene was PCR amplified from wild isolates and references strains using the primers 27f (5-’CTGGGATCCATTTACTCGAGAGTTTGATCCTGGCTCAG-3’) and 1490r (5’-GGTTCCCCTAAGCTTACCTTGTTACGACTTC-3’)^64^ at the following conditions: 5 min at 95 °C, 30 cycles with 1 min at 95 °C, 2.5 min at 58 °C and 2 min at 72 °C and 5 min of final extension at 72 °C. PCR amplicons were digested with *Mse*I restriction enzyme for 3 h according to manufacturer’s instructions and the resulting DNA restriction fragments were separated by electrophoresis in a 2% (w/v) agarose gel with ethidium bromide (0.5 mg/mL) in 0.5X Tris–borate-EDTA (pH 8.0) buffer at 90 V for 90 min and visualized under a UV source. Each gel was documented with a GelDoc apparatus (Biometra, Göttingen, Germany). When required, 16S rDNA amplicons were also digested with *EcoR*I^27^. For all the investigated bacterial strains (reference and wild), restriction fragment sizes were measured (in bp) by comparison with the GeneRuler 100 bp Plus bp DNA Ladder (Thermo Scientific, Waltham, MA, USA).

At least one amplicon for each 16S-ARDRA profile was Sanger sequenced with both amplification primers by BMR Genomics (Padua, Italy). Trimmed sequences were annotated by MegaBLAST search against the NCBI-Refseq database using minimum cutoff values of 98% identity. Phylogenetic placement of query and reference sequences was conducted in MEGAX^65^ (Biomatters, Auckland, NZ) and analyzed using Neighbor joining method^66^. The rate variation among sites was modeled with a gamma distribution (shape parameter = 1)^67^. The branches of the inferred unrooted tree were assayed using bootstrap analysis with 1000 replicates^68^. The sequences obtained from bacterial cultures were deposited in the GenBank NCBI database with the accession numbers ON755074-ON755086. Genotyping of isolates was carried out by (GTG)_5_ repetitive bacterial DNA elements PCR (rep-PCR) as previously reported^63^. All trees were visualized using Interactive Tree of Life (ITOL)^69^.

### Total microbial DNA extraction and quantification

Total bacterial DNAs were extracted with the Dneasy PowerSoil DNA kit (Qiagen, Valencia, CA, USA). Briefly, NWS samples were thawed on ice, divided into five aliquots of 1 mL each and centrifuged at 16,000×*g* for 5 min. Cells were resuspended into 800 μL of CD1 buffer, added with 200 μL of zirconium beads (0.1 mm) and incubated at 65 °C for 10 min. After vortexing with a TissueLyzer (Qiagen) at 25 Hz for 10 min, 550 μL of lysate were added with 250 μL of CD2 buffer and DNA was extracted according to the manufacturer’s instructions. All DNA samples were diluted into 120 μL and checked for integrity by electrophoresis in a 1.5% (w/v) agarose gel, while quantity was verified by absorbance measurements at 260 and 280 nm using a Nanodrop (NanoDrop™ 2000, Thermo Fisher Scientific, Waltham, Massachusetts, USA), and its integrity was verified by electrophoresis on 1.5% agarose gels. DNA was stored at − 80 °C before further processing.

### Illumina sequencing and data processing

Total DNA samples were used as a template for the preparation of 16S rRNA gene amplicon libraries following standard Illumina library preparation procedure^70^. Briefly, the V3-V4 hypervariable regions of the 16S rRNA gene were initially amplified using a universal pair of primer: 341F (5′-CCTACGGGNBGCASCAG-3′) and 805R (5′-GACTACNVGGGTATCTAATCC-3′) resulting in an amplicon length of ~ 464 bp^71^. A second PCR was then carried out to attach dual Illumina barcode indices and adapters using the Nextera XT library preparation kit (Illumina, San Diego, CA). PCRs products were purified and normalized using a SequalPrep Normalization Plate and sequenced through 300 × 2 bp paired-end sequencing on an Illumina MiSeq V3 platform (Illumina, San Diego, CA, USA) at BMR Genomics (Padua, Italy).

Microbial sequences were processed in the Quantitative Insights into Microbial Ecology 2 (QIIME 2) bioinformatics platform, version qiime2-2021^72^. Primers were removed using the q2-cutadapt plugin. Later, paired-end sequences were subjected to quality control including denoising, merging, and chimera removal using the DADA2 plugin^73^ implemented in QIIME 2 (dada2 denoise-paired with the following parameters trunc_len_f:260, trunc_len_r:245). The resulting table of amplicon sequence variants (ASV)^74^ was subsequently filtered at 0.001% to remove singletons and very rare ASVs. Taxonomic classification of ASVs was carried out with q2-feature-classifier plugin^75^ using trained OTUs at 99% from Silva database version 138^76^. The taxonomic assignment of poorly classified ASVs were manually verified using NCBI blastn^77^. Samples were rarefied to 23,726 sequences by random subsampling in QIIME2 before downstream alpha and beta diversity analyses. No samples were excluded by the rarefaction step.

### Ecological and statistical analysis

Statistical computing and graphical generation were performed using the R programing environment unless otherwise indicated (r-project.org), while alpha and beta diversity analyses were done using the various tools of QIIME2 diversity plugin. To evaluate alpha diversity, for each sample we computed various indices including observed features, Shannon, evenness, and Faith’s phylogenetic diversity (Faith's PD). The Kruskal–Wallis test was used as a non-parametric statistical test to test pairwise differences (https://docs.qiime2.org/2022.2/plugins/available/diversity/alpha-group-significance/). The distance matrices of the main beta diversity metrics (Bray–Curtis, Jaccard, weighted Unifrac, and unweighted Unifrac) and their corresponding principal coordinate analysis (PCoA) were also calculated, in order to investigate the dissimilarity in bacterial communities between dairy farms. Statistical analyses of beta diversity were conducted using the PERMANOVA test with 999 permutations (beta-group-significance). Beta diversity PCoA were projected onto 2D ordination plots by using ggplot2 and ellipses were drawn for each pond around 95% confidence intervals, assuming a multivariate normal distribution, applying the stat_ellipse function included in the same package. Differential abundance of taxa was tested at different levels (family, genus, species, and ASV) using ANCOM^30^ plugin implemented in Qiime2. To avoid errors during data normalization step, pseudocounts were added to ASV table using “qiime composition add-pseudocount” before running ANCOM.

Then we applied Partial Least Squares Discriminant Analysis (PLS-DA) with mixOmics^78^ to divide the samples into different predefined type-H and D and finally sparse PLS-DA (sPLS-DA) was used to select the most discriminative features able to separate the two main groups.

Parametric statistics (one-way ANOVA and related *post-hoc* tests) on chemical data and Spearman correlation coefficient analysis with a two-sided 95% confidence interval were computed using GraphPad Prism 8 software (San Diego, CA, USA).

The overall level of significance was set at *p* < 0.05.

### Functional analysis

Functional abundances were inferred to frequency filtered ASVs table using PICRUSt2 pipeline (v2.4.1)^31^. Subsequently, three tools available in Bioconductor R package^79^ (ALDEx2 1.26.0^32^, ANCOM-BC 1.4.0^33^ and MaAsLin2 1.8.0^34^) were applied to perform the differential analysis of PICRUSt2-predicted pathways relative abundances and to identify the most characterizing pathways in the two main groups. In all methods a minimum prevalence cutoff of 0.1 was set as an analysis parameter. Moreover, since ALDEx2 uses centered log-ratio (clr) transformation and PICRUSt2 sometimes outputs non-integers values, it was necessary to round values (https://forum.qiime2.org/t/aldex2-error-when-processing-picrust2-output/14104). Significant values for ALDEx2 were filtered considering a q-value cutoff of 0.1 on the Expected Benjamini-Hochberg (BH) corrected P value of Wilcoxon test. A *q*-value cutoff of 0.1 on the adjusted p-value was applied to retain only most significant results also from ANCOM-BC and MaAsLin2 outputs. A consensus method was finally applied to the results to mark as significant only those pathways that were selected by all the three methods. Visual representation of the top 15 most significant pathways was created using the pheatmap method of the pheatmap R package v.1.0.12^80^.

### Milk acidification assays

Every strain was precultured in 35 mL of the corresponding growth medium until the late exponential phase at 42 °C under anaerobiosis for *L. delbrueckii* and *L. helveticus* and under aerobiosis for *St. thermophilus* tester strains, respectively. After assessment of optical density at 600 nm, cells washed with saline water (9 g/L NaCl) and used to inoculate 50 mL of UHT skimmed milk in sterile glass bottles at the final concentration of 2 × 10^7^ CFU/mL as monocultures. In coculture (*L. delbrueckii* × *St. thermophilus*) and triculture (*L. delbrueckii* × *St. thermophilus* × *L. helveticus*) experiments the strains were mixed each other at the final concentration of 2 × 10^7^ CFU/mL. Milk samples were incubated at 42 °C and fermentation trials were monitored in triplicates as pH decrease over time with a pH meter (XS Instruments, Carpi, Italy). Uninoculated milk was used as negative control. Ten mL aliquot of a freshly prepared NWS was sampled from a dairy farm in the PDO production area of PR cheese and immediately transferred to the laboratory and used to inoculate milk as positive control. NWS cells concentration was assessed a Bürker glass chamber to inoculate UHT skimmed milk with 2 × 10^7^ cell/mL.

## Supplementary Information

Supplementary Figures.

Supplementary Tables.

## Data Availability

The raw data associated to this study are available in the NCBI Sequence Read Archive (SRA) database under the BioProject number PRJNA857875. Other data are available by request to lisa.solieri@unimore.it.

## References

[1] Smid EJ (2014). Functional implications of the microbial community structure of undefined mesophilic starter cultures. Microb. Cell Factories.

[2] Stadhouders J, Leenders GJM (1984). Spontaneously developed mixed-strain cheese starters. Their behaviour towards phages and their use in the Dutch cheese industry. Neth. Milk Dairy J..

[3] Fox PF, Cogan TM, Guinee TP, McSweeney PLH, Fox PF, Cotter PD, Everett DW (2017). Factors that affect the quality of cheese. Cheese: Chemistry, Physics and Microbiology.

[4] De Vos WM (2011). Systems solutions by lactic acid bacteria: From paradigms to practice. Microb. Cell Fact..

[5] Sieuwerts S, de Bok FA, Hugenholtz J, van Hylckama Vlieg JE (2008). Unraveling microbial interactions in food fermentations: From classical to genomics approaches. Appl. Environ. Microbiol..

[6] Erkus O (2013). Multifactorial diversity sustains microbial community stability. ISME J..

[7] Somerville V (2019). Long-read based de novo assembly of low-complexity metagenome samples results in finished genomes and reveals insights into strain diversity and an active phage system. BMC Microbiol..

[8] Mayo B, Rodríguez J, Vázquez L, Flórez AB (2021). Microbial Interactions within the cheese ecosystem and their application to improve quality and safety. Foods.

[9] Casey E (2022). Needle in a whey-stack: PhRACS as a discovery tool for unknown phage-host combinations. MBio.

[10] Giraffa G (2021). The microbiota of Grana Padano cheese. A review. Foods.

[11] Cocconcelli PS, Parisi MG, Senini L, Bottazzi V (1997). Use of RAPD and 16S rDNA sequencing for the study of *Lactobacillus* population dynamics in natural whey culture. Lett. Appl. Microbiol..

[12] Coppola R (2000). Microbiological characteristics of Parmigiano Reggiano cheese during the cheesemaking and the first months of the ripening. Lait.

[13] Gatti M, Lazzi C, Rossetti L, Mucchetti G, Neviani E (2003). Biodiversity in *Lactobacillus helveticus* strains present in natural whey starter used for Parmigiano Reggiano cheese. J. Appl. Microbiol..

[14] Gatti M, Trivisano C, Fabrizi E, Neviani E, Gardini F (2004). Biodiversity among *Lactobacillus helveticus* strains isolated from different natural whey starter cultures as revealed by classification trees. Appl. Environ. Microbiol..

[15] Bottari B, Santarelli M, Neviani E, Gatti M (2004). Natural whey starter for Parmigiano Reggiano: Culture-independent approach. J. Appl. Microbiol..

[16] Bertani G, Levante A, Lazzi C, Bottari B, Gatti M, Neviani E (2020). Dynamics of a natural bacterial community under technological and environmental pressures: The case of natural whey starter for Parmigiano Reggiano cheese. Food Res. Int..

[17] Lazzi C, Rossetti L, Zago M, Neviani E, Giraffa G (2004). Evaluation of bacterial communities belonging to natural whey starters for Grana Padano cheese by length heterogeneity-PCR. J. Appl. Microbiol..

[18] Ercolini D, Frisso G, Mauriello G, Salvatore F, Coppola S (2008). Microbial diversity in natural whey cultures used for the production of Caciocavallo Silano PDO cheese. Int. J. Food Microbiol..

[19] De Filippis F, La Storia A, Stellato G, Gatti M, Ercolini D (2014). A selected core microbiome drives the early stages of three popular Italian cheese manufactures. PLoS ONE.

[20] Levante A (2021). How new molecular approaches have contributed to shedding light on microbial dynamics in Parmigiano Reggiano cheese. Curr. Opin. Food Sci..

[21] Zotta T, Ricciardi A, Condelli N, Parente E (2022). Metataxonomic and metagenomic approaches for the study of undefined strain starters for cheese manufacture. Crit. Rev. Food Sci. Nutr..

[22] Gänzle MG (2015). Lactic metabolism revisited: Metabolism of lactic acid bacteria in food fermentations and food biotechnology. Curr. Opin. Food Sci..

[23] Torino MI, Taranto MP, FontdeValdez G (2005). Citrate catabolism and production of acetate and succinate by *Lactobacillus helveticus* ATCC 15807. Appl. Microbiol. Biotechnol..

[24] Warnecke T, Gill TR (2005). Organic acid toxicity, tolerance, and production in *Escherichia coli* biorefining applications. Microb. Cell Fact..

[25] Coloretti F (2017). Detection and identification of yeasts in natural whey starter for Parmigiano Reggiano cheese-making. Int. Dairy J..

[26] Martini S (2021). Characterization of yeasts isolated from Parmigiano Reggiano cheese natural whey starter: From spoilage agents to potential cell factories for whey valorization. Microorganisms.

[27] Giraffa G, De Vecchi P, Rossetti L (1998). Identification of *Lactobacillus delbrueckii* subspecies *bulgaricus* and subspecies *lactis* dairy isolates by amplified rDNA restriction analysis. J. Appl. Microbiol..

[28] Fornasari ME, Rossetti L, Carminati D, Giraffa G (2006). Cultivability of *Streptococcus thermophilus* in Grana Padano cheese whey starters. FEMS Microbiol. Lett..

[29] Stackebrandt E, Ebers J (2006). Taxonomic parameters revisited: Tarnished gold standards. Microbiol. Today.

[30] Mandal S (2015). Analysis of composition of microbiomes: A novel method for studying microbial composition. Microbial Ecol. Health Dis..

[31] Douglas GM (2020). PICRUSt2: An improved and extensible approach for metagenome inference. Nat. Biotechnol..

[32] Fernandes AD, Macklaim JM, Linn TG, Reid G, Gloor GB (2013). ANOVA-like differential expression (ALDEx) analysis for mixed population RNA-Seq. PLoS ONE.

[33] Lin H, Peddada SD (2020). Analysis of compositions of microbiomes with bias correction. Nat. Commun..

[34] Mallik H (2021). Multivariable association discovery in population-scale meta-omics studies. PLoS Comput. Biol..

[35] Tofalo R (2014). The predominance, biodiversity and biotechnological properties of *Kluyveromyces marxianus* in the production of Pecorino di Farindola cheese. Int. J. Food Microbiol..

[36] Parente E (2016). Microbial community dynamics in thermophilic undefined milk starter cultures. Int. J. Food Microbiol..

[37] Somerville V (2022). Functional strain redundancy and persistent phage infection in Swiss hard cheese starter cultures. ISME J..

[38] Courtin P, Monnet V, Rul F (2022). Cell-wall proteinases PrtS and PrtB have a different role in *Streptococcus thermophilus*/*Lactobacillus bulgaricus* mixed cultures in milk. Microbiology.

[39] Yamauchi R, Maguin E, Horiuchi H, Hosokawa M, Sasaki Y (2019). The critical role of urease in yogurt fermentation with various combinations of *Streptococcus thermophilus* and *Lactobacillus delbrueckii* ssp. bulgaricus. J. Dairy Sci..

[40] Moser A, Schafroth K, Meile L, Egger L, Badertscher R, Irmler S (2018). Population Dynamics of *Lactobacillus helveticus* in Swiss Gruyère-Type Cheese manufactured with natural whey cultures. Front. Microbiol..

[41] Hebert EM, Raya RR, De Giori GS (2000). Nutritional requirements and nitrogen-dependent regulation of proteinase activity of *Lactobacillus helveticus* CRL 1062. Appl. Environ. Microbiol..

[42] Slattery L, O'Callaghan J, Fitzgerald GF, Beresford T, Ross RP (2010). Invited review: *Lactobacillus helveticus*: A thermophilic dairy starter related to gut bacteria. J. Dairy Sci..

[43] Daly DFM, McSweeney PLH, Sheehan JJ (2009). Split defect and secondary fermentation in Swiss-type cheeses: A review. Dairy Sci. Technol..

[44] Mancini A (2021). Massive survey on bacterial–bacteriophages biodiversity and quality of natural whey starter cultures in Trentingrana cheese production. Front. Microbiol..

[45] Turner K, Martley F (1983). Galactose fermentation and classification of thermophilic lactobacilli. Appl. Environ. Microbiol..

[46] Smit G, Smit BA, Engels WJ (2005). Flavour formation by lactic acid bacteria and biochemical flavour profiling of cheese products. FEMS Microbiol. Rev..

[47] Santarelli M (2013). Variability of lactic acid production, chemical and microbiological characteristics in 24-hour Parmigiano Reggiano cheese. Dairy Sci. Technol..

[48] Jousset A, Schulz W, Scheu S, Eisenhauer N (2011). Intraspecific genotypic richness and relatedness predict the invasibility of microbial communities. ISME J.

[49] Heuer H, Abdo Z, Smalla K (2008). Patchy distribution of flexible genetic elements in bacterial populations mediates robustness to environmental uncertainty. FEMS Microbiol. Ecol..

[50] Dobrindt U, Hochhut B, Hentschel U, Hacker J (2004). Genomic islands in pathogenic and environmental microorganisms. Nat. Rev. Microbiol..

[51] Rodriguez-Valera F (2009). Explaining microbial population genomics through phage predation. Nat. Rev. Microbiol..

[52] Thingstad TF (2000). Elements of a theory for the mechanisms controlling abundance, diversity, and biogeochemical role of lytic bacterial viruses in aquatic systems. Limnol. Oceanogr..

[53] Fortina MG, Rossi P, Mora D, Parini C, Neviani E (1996). Slow milk coagulating variants of *Lactobacillus helveticus*. Folia Microbiol..

[54] Miyamoto M (2015). Distinctive proteolytic activity of cell envelope proteinase of *Lactobacillus helveticus* isolated from airag, a traditional Mongolian fermented mare's milk. Int. J. Food Microbiol..

[55] Sadat-Mekmene L (2011). Simultaneous presence of PrtH and PrtH2 proteinases in *Lactobacillus helveticus* strains improves breakdown of the pure αS1-casein. Appl. Environ. Microbiol..

[56] Hebert EM, De Giori GS, Raya RR (2001). Isolation and characterization of a slowly milk-coagulating variant of *Lactobacillus helveticus* deficient in purine biosynthesis. Appl. Environ. Microbiol..

[57] Schmid M (2018). Comparative genomics of completely sequenced *Lactobacillus helveticus* genomes provides insights into strain-specific genes and resolves metagenomics data down to the strain level. Front. Microbiol..

[58] Morris JJ, Lenski RE, Zinser ER (2012). The Black Queen hypothesis: Evolution of dependencies through adaptive gene loss. MBio.

[59] Bachmann H (2011). High local substrate availability stabilizes a cooperative trait. ISME J..

[60] Giraffa G, Mucchetti G, Neviani E (1996). Interactions among thermophilic lactobacilli during growth in cheese whey. J. Appl. Bacteriol..

[61] Coloretti F (2016). Whey starter addition during maturation of evening milk: Effects on some characteristics of cheese milk and Parmigiano-Reggiano cheese. Dairy Sci. Technol..

[62] Terzaghi BE, Sandine W (1975). Improved medium for lactic streptococci and their bacteriophages. Appl. Microbiol..

[63] Tagliazucchi D (2020). Cultivable non-starter lactobacilli from ripened Parmigiano Reggiano cheeses with different salt content and their potential to release anti-hypertensive peptides. Int. J. Food Microbiol..

[64] Lane DJ, Stackebrandt E, Goodfellow M (1991). 16S/23S rRNA sequencing. Nucleic Acid Techniques in Bacterial Systematics.

[65] Kumar S, Stecher G, Li M, Knyaz C, Tamura K (2018). MEGA X: Molecular evolutionary genetics analysis across computing platforms. Mol. Biol. Evol..

[66] Saitou N, Nei M (1987). The neighbor-joining method: A new method for reconstructing phylogenetic trees. Mol. Biol. Evol..

[67] Kimura M (1980). A simple method for estimating evolutionary rate of base substitutions through comparative studies of nucleotide sequences. J. Mol. Evol..

[68] Felsenstein J (1985). Confidence limits on phylogenies: An approach using the bootstrap. Evolution.

[69] Letunic I, Bork P (2011). Interactive Tree of Life v2: Online annotation and display of phylogenetic trees made easy. Nucleic Acids Res..

[70] Illumina Inc. *16S Metagenomic Sequencing Library Preparation*. https://support.illumina.com/documents/documentation/chemistry_documentation/16s/16s-metagenomic-library-prep-guide-15044223-b.pdf. (2020)

[71] Takahashi S, Tomita J, Nishioka K, Hisada T, Nishijima M (2014). Development of a prokaryotic universal primer for simultaneous analysis of Bacteria and Archaea using next-generation sequencing. PLoS ONE.

[72] Bolyen E (2019). Reproducible, interactive, scalable and extensible microbiome data science using QIIME 2. Nat. Biotechnol..

[73] Callahan BJ (2016). DADA2: High-resolution sample inference from Illumina amplicon data. Nat. Methods.

[74] Callahan BJ, McMurdie PJ, Holmes SP (2017). Exact sequence variants should replace operational taxonomic units in marker-gene data analysis. ISME J..

[75] Bokulich NA (2018). Optimizing taxonomic classification of marker-gene amplicon sequences with QIIME 2’s q2-feature-classifier plugin. Microbiome.

[76] Quast C (2012). The SILVA ribosomal RNA gene database project: Improved data processing and web-based tools. Nucleic Acids Res..

[77] Edgar RC (2010). Search and clustering orders of magnitude faster than BLAST. Bioinformatics.

[78] Rohart F, Gautier B, Singh A, Lê Cao KA (2017). mixOmics: An R package for ‘omics feature selection and multiple data integration. PLoS Comput. Biol..

[79] Morgan, M. *BiocManager: Access the Bioconductor Project Package Repository*. *R package version 1.30.16* (2018).

[80] Kolde R. *pheatmap: Pretty Heatmaps. R package version 1.0. 12. CRAN. R-project. org/package= pheatmap*. (2019).

