# Supplementary material for: Insights on the bacterial composition of Parmigiano Reggiano Natural Whey Starter by a culture-dependent and 16S rRNA metabarcoding portrait
Source: Sci Rep. 2022 Oct 15;12:17322. doi: 10.1038/s41598-022-22207-y (PMC9569347; doi:10.1038/s41598-022-22207-y)
Supplement: Supplementary file 1 — Supplementary Figures. [file 41598_2022_22207_MOESM1_ESM.pdf]

Insights on the bacterial composition of Parmigiano Reggiano Natural Whey Starter by a culture-dependent and 16S rRNA metabarcoding portrait

Laura Sola[1], Emanuele Quadu[1], Elena Bortolazzo[2], Loris Bertoldi[3], Cinzia L Randazzo[4,5], Valentina Pizzamiglio[6], Lisa Solieri[1]*

[1]Department of Life Sciences, University of Modena and Reggio Emilia, Reggio Emilia, 42122, Italy

[2]Centro Ricerche Produzioni Animali, Reggio Emilia, 42121, Italy

[3]BMR Genomics srl, Padua, 35131, Italy

[4]Department of Agriculture, Food and Environment, University of Catania, Catania, 95123 Italy

[5]ProBioEtna srl, Catania, 95123 Italy

[6]Consorzio del Formaggio Parmigiano Reggiano, 42124 Reggio Emilia, Italy

*Corresponding author: lisa.solieri@unimore.it

Supplementary Figures

Supplementary Figure S1. Dendrogram obtained through UPGMA analysis of the (GTG)$_5$ rep-PCR profiles of 57 strains isolated from PR NWS samples using the Pearson's correlation coefficient. The similarity value above 88% was used to discriminate biotypes numbered from S1 to S14. Each knot shows the length of the arms. The colors have been attributed to species, as follows: blue, *L. helveticus*; orange, *L. delbrueckii* subsp. *lactis*; yellow, *St. thermophilus*; grey, *S capitis*. The tree was visualized with ITOL [1]. 9

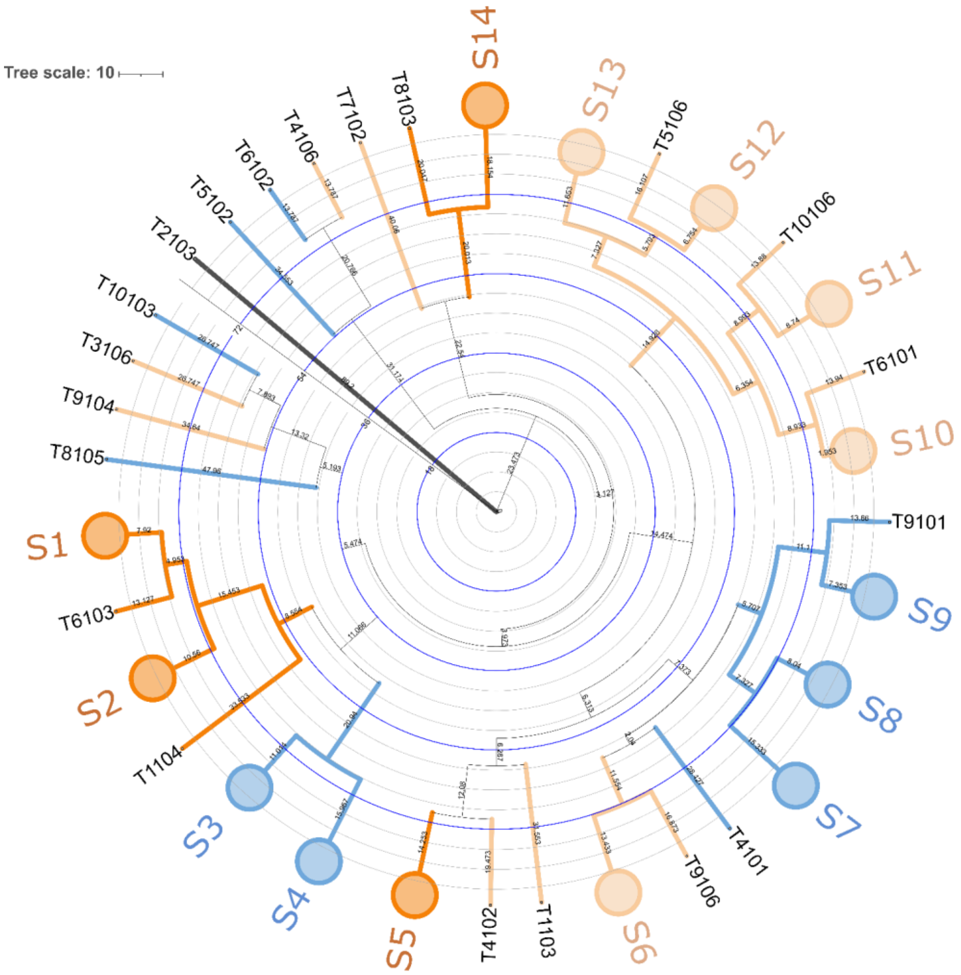

Supplementary Figure S2. **Rarefaction curves showing the number of observed features (A) and Shannon index (B) as functions of the number of sequenced reads in PR NWS samples.** The minimum sample size (23,726) achieved over the studied NWS samples was used.

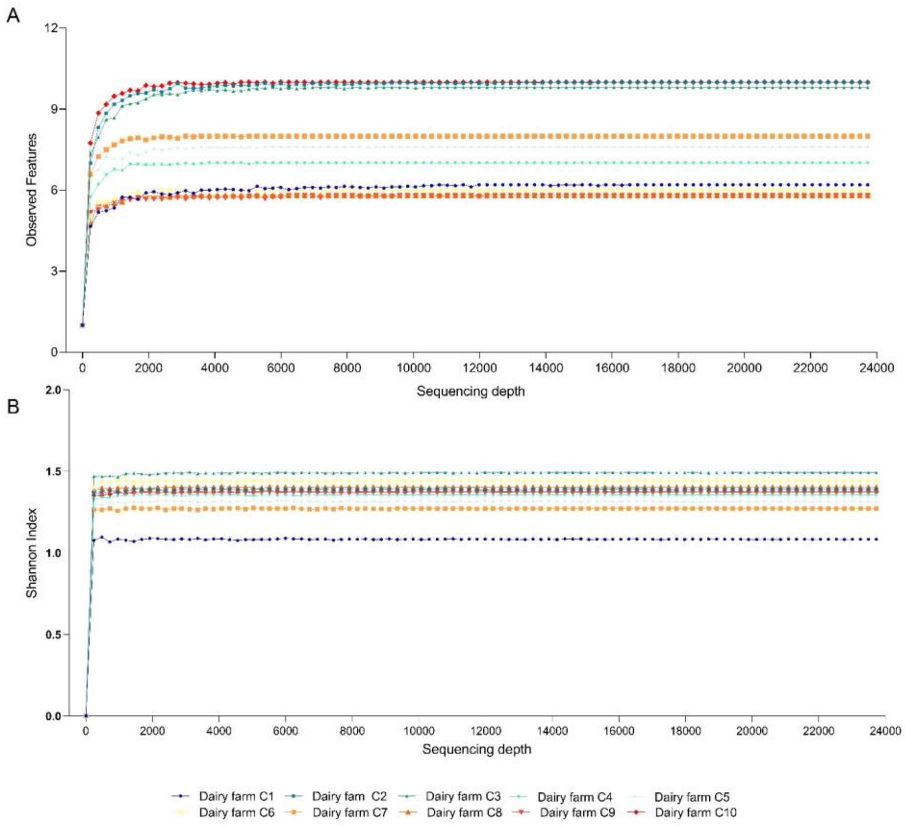

Supplementary Reference

1. Letunic, I. & Bork, P. Interactive Tree of Life v2: online annotation and display of phylogenetic trees made easy. *Nucleic Acids Res*. W475–W478 (2011).
