# Supplementary material for: Insights on the bacterial composition of Parmigiano Reggiano Natural Whey Starter by a culture-dependent and 16S rRNA metabarcoding portrait
Source: Sci Rep. 2022 Oct 15;12:17322. doi: 10.1038/s41598-022-22207-y (PMC9569347; doi:10.1038/s41598-022-22207-y)
Supplement: Supplementary file 2 — Supplementary Tables. [file 41598_2022_22207_MOESM2_ESM.pdf]

Insights on the bacterial composition of Parmigiano Reggiano Natural Whey Starter by a culture-dependent and 16S rRNA metabarcoding portrait

Laura Sola[1], Emanuele Quadu[1], Elena Bortolazzo[2], Loris Bertoldi[3], Cinzia L Randazzo[4,5], Valentina Pizzamiglio[6], Lisa Solieri[1]*

[1]Department of Life Sciences, University of Modena and Reggio Emilia, Reggio Emilia, 42122, Italy

[2]Centro Ricerche Produzioni Animali, Reggio Emilia, 42121, Italy

[3]BMR Genomics, Padua, 35131, Italy

[4]Department of Agriculture, Food and Environment, University of Catania, Catania, 95123 Italy

[5]ProBioEtna srl, Catania, 95123 Italy

[6]Consorzio del Formaggio Parmigiano Reggiano, 42124 Reggio Emilia, Italy

[*]Corresponding author: lisa.solieri@unimore.it

Supplementary Tables

Supplementary Table S1. Raw data of physicochemical and microbial counts. Viable counts were expressed as mean Log_{10} CFU/mL.

Sample	Physicochemical data			Organic acids (g/L)				Viable counts				
	pH	°SH/50 mL	Δ°SH/50 mL	Succinic acid	Citrate	Acetic acid	Lactic acid	MRS 42°C; anaerobiosis	M17-SSW 42°C; anaerobiosis	M17-SSW 42°C aerobiosis	YPDA 28°C	YPLA 42°C
**C1**	3.36	30.2	1.79	0.206 ± 0.004	0.350 ± 0.003	0.276 ± 0.003	13.511 ± 0.197	8.367 ± 0.174	8.374 ± 0.170	8.234 ± 0.029	4.796 ± 0.192	4.100 ± 0.075
**C2**	3.29	29.9	1.19	0.206 ± 0.013	0.289 ± 0.016	0.226 ± 0.001	13.090 ± 0.512	7.820 ± 0.139	7.267 ± 0.047	6.828 ± 0.107	1.841 ± 0.088	1.349 ± 0.494
**C3**	3.46	31.4	2.15	0.214 ± 0.007	0.306 ± 0.008	0.289 ± 0.001	13.205 ± 0.656	7.717 ± 0.105	7.760 ± 0.185	7.753 ± 0.107	2.958 ± 0.042	2.768 ± 0.119
**C4**	3.41	30.1	2.80	0.197 ± 0.004	0.360 ± 0.045	0.319 ± 0.002	13.446 ± 1.680	7.545 ± 0.239	8.118 ± 0.085	7.804 ± 0.084	2.382 ± 0.180	2.159 ± 0.143
**C5**	3.57	27.3	1.98	0.179 ± 0.009	0.305 ± 0.045	0.212 ± 0.003	11.953 ± 0.039	7.474 ± 0.104	8.036 ± 0.094	8.095 ± 0.103	3.681 ± 0.335	3.503 ± 0.341
**C5**	3.45	30.5	2.51	0.228 ± 0.010	0.365 ± 0.040	0.288 ± 0.009	15.292 ± 0.039	7.768 ± 0.187	7.548 ± 0.172	7.484 ± 0.267	4.000 ± 0.201	1.167 ± 0.301
**C7**	3.27	32.8	2.97	0.211 ± 0.010	0.333 ± 0.031	0.409 ± 0.029	12.593 ± 0.684	8.679 ± 0.134	7.204 ± 0.267	6.651 ± 0.234	3.995 ± 0.083	3.875 ± 0.036
**C8**	3.40	33.4	2.95	0.205 ± 0.010	0.337 ± 0.014	0.325 ± 0.034	15.069 ± 1.220	8.473 ± 0.194	7.982 ± 0.137	7.856 ± 0.041	2.318 ± 0.186	1.000 ± 0.001
**C9**	3.48	31.5	3.37	0.156 ± 0.004	0.260 ± 0.004	0.233 ± 0.033	13.872 ± 2.362	8.701 ± 0.116	8.247 ± 0.065	7.970 ± 0.081	1.900 ± 0.077	1.759 ± 0.720
**C10**	3.42	28.6	2.07	0.194 ± 0.002	0.267 ± 0.012	0.450 ± 0.015	11.720 ± 0.464	7.762 ± 0.062	7.590 ± 0.131	7.165 ± 0.085	1.452 ± 0.213	1.540 ± 0.337

Supplementary Table S2. Phenotype characterization and 16S ARDRA patterns of thermophilic isolates from PR NWS samples. Fragments lower than 70 bp were omitted. Strains in bold and underlined were submitted to 16S rRNA gene Sanger sequencing.

Sample	Growth conditions	Codice ceppi	Morphology	Gram staining	Catalase reaction	16S-ARDRA (bp)	
						***Mse*I**	***EcoR*I**
C1	MRS (pH 4.5); 42°C; anaerobiosis	**<u>T1101</u>**, T1102	Rods in long chain	+	-	650-390-260-140-90	na
	M17-SSW; 42°C; anaerobiosis	T1103	Cocci in pairs or short chain	+	-	600-260-250-200-140	na
		**<u>T1104</u>**	Rods in long chain	+	-	900-260-160-150-100	1550
	M17-SSW; 42°C; aerobiosis	T1105, T1106	Cocci in pairs or short chain	+	-	600-260-250-200-140	na
C2	MRS (pH 4.5); 42°C; anaerobiosis	T2101, T2102	Rods in long chain			650-390-260-140-90	na
	M17-SSW; 42°C; anaerobiosis	**<u>T2103</u>**	Cocci in pairs	+	+	590-390-290-140-90	na
		**<u>T2104</u>**, T2106	Rods in long chain	+	-	900-260-160-150-100	1550
	M17-SSW; 42°C; aerobiosis	T2105	Cocci in pairs or short chain	+	-	600-260-250-200-140	na
C3	MRS (pH 4.5); 42°C; anaerobiosis	T3101, T3103	Rods in long chain	+	-	650-390-260-140-90	na
	M17-SSW; 42°C; anaerobiosis	T3104, T3105, T3106	Cocci in pairs or short chain	+	-	600-260-250-200-140	na
C4	MRS (pH 4.5); 42°C; anaerobiosis	T4101	Rods in long chain	+	-	650-390-260-140-90	na
		T4102, T4104, T4105, T4106,	Cocci in pairs or short chain	+	-	600-260-250-200-140	na
	M17-SSW; 42°C; anaerobiosis	**T4103**	Rods in long chain			900-260-160-150-100	1550
C5	MRS (pH 4.5); 42°C; anaerobiosis	T5101, T5102	Rods in long chain	+	-	650-390-260-140-90	na
	M17-SSW; 42°C; anaerobiosis	T5103, T5104, **<u>T5105</u>**	Rods in long chain	+	-	900-260-160-150-100	1550
	M17-SSW; 42°C; aerobiosis	T5106	Cocci in pairs or short chain	+	-	600-260-250-200-140	na
C6	MRS (pH 4.5); 42°C; anaerobiosis	T6101, T6105	Cocci in pairs or short chain	+	-	600-260-250-200-140	na

		T6102, T6104 T6106	Rods in long chain	+	-	650-390-260-140-90	na
	M17-SSW; 42°C; anaerobiosis	**T6103**	Rods in long chain	+	-	900-260-160-150-100	1550
C7	MRS (pH 4.5); 42°C; anaerobiosis	T7102 T7105	Cocci in pairs or short chain	+	-	600-260-250-200-140	na
	M17-SSW; 42°C; anaerobiosis	T7103, T7104, T7106	Rods in long chain	+	-	900-260-160-150-100	1550
C8	MRS (pH 4.5); 42°C; anaerobiosis	**T8102**, T8105	Rods in long chain	+	-	650-390-260-140-90	na
	M17-SSW; 42°C; anaerobiosis	T8103	Rods in long chain	+	-	900-260-160-150- 0	1550
		T8104, **T8106**	Cocci in pairs or short chain	+	-	600-260-250-200-140	na
C9	MRS (pH 4.5); 42°C; anaerobiosis	**T9101**, T9102, T9103	Rods in long chain	+	-	650-390-250-140-90	na
	M17-SSW; 42°C; anaerobiosis	T9104, T9105, T9106	Cocci in pairs or short chain	+	-	600-260-250-200-140	na
C10	MRS (pH 4.5); 42°C; anaerobiosis	T10101, **T10102**, T10103	Rods in long chain	+	-	650-390-250-140-90	na
	M17-SSW; 42°C; anaerobiosis	**T10104**	Rods in long chain	+	-	900-260-160-150-100	1550
	M17-SSW; 42°C; aerobiosis	**T10105**, T10106	Cocci in pairs or short chain	+	-	600-260-250-200-140	na

Abbreviations: na, not applicable.

Supplementary Table S3. Dairy farm localization and features of the cheese-making and NWS production processes.

Dairy farm	Province	Altitude area	N. of milk-supplying farms	n. of vats	Curd cooking temperature °C	Kind of fermentation unit	N. of fermentation units	NWS production capacity (L)
C1	Reggio Emilia	Plain	9	30	55.0	automatic	2	140
C2	Reggio Emilia	Plain	1	11	54.5	mixed	1	100
C3	Reggio Emilia	Plain	12	24	55.3	mixed	4	145
C4	Mantova	Plain	27	61	54.6	automatic	2	260
C5	Reggio Emilia	Plain	18	42	54.6	mixed	4	170
C6	Parma	Plain	8	20	55.0	mixed	2	72
C7	Parma	Plain	1	10	54.7	automatic	3	76
C8	Reggio Emilia	Plain	14	27	54.7	automatic	2	120
C9	Modena	Mountain	20	19	54.6	automatic	3	120
C10	Modena	Plain	40	49	55.0	mixed	2	280

Supplementary Table S4. Temperature curves trends during NWS fermentative production.

**Dairy ID**	C1	C2	C3	C4	C5	C6	C7	C8	C9	C10
**Cluster**	D	H	D	D	D	D	H	D	H	H
**Total fermentation time(min)**	760	700	1100	933	1140	830	565	990	820	810
**Temperature range**	**% relative retention time ***									
45-48°C	76	51	16	12	4	31	62	9	27	16
40-45°C	24	49	49	44	47	55	38	48	44	70
35-40°C	0	0	35	38	32	13	0	42	29	14
35-40°C	0	0	0	6	18	0	0	0	0	0
